# Effects of antiplatelet therapy after stroke due to intracerebral haemorrhage (RESTART): a randomised, open-label trial

**DOI:** 10.1016/S0140-6736(19)30840-2

**Published:** 2019-06-29

**Authors:** RESTART Collaboration, Rustam Al-Shahi Salman, MS Dennis, PAG Sandercock, CLM Sudlow, JM Wardlaw, WN Whiteley, GD Murray, J Stephen, DE Newby, N Sprigg, DJ Werring, PM White, Colin Baigent, Daniel Lasserson, Frank Sullivan, Johanna Carrie, Javier Rojas, Shannon Amoils, John Bamford, Jane Armitage, Gabriel Rinkel, Gordon Lowe, Jonathan Emberson, Karen Innes, Lynn Dinsmore, Jonathan Drever, Carol Williams, David Perry, Connor McGill, David Buchanan, Allan Walker, Aidan Hutchison, Christopher Matthews, Ruth Fraser, Aileen McGrath, Ann Deary, Rosemary Anderson, Pauli Walker, Christian Hansen, Richard Parker, Aryelly Rodriguez, MR Macleod, Thomas Gattringer, Jeb Palmer, Eleni Sakka, Jennifer Adil-Smith, David Minks, Dipayan Mitra, Priya Bhatnagar, Johannes du Plessis, Yogish Joshi, Christine Lerpiniere, Richard O'Brien, Seona Burgess, Gillian Mead, Ruth Paulton, Fergus Doubal, Katrina McCormick, Neil Hunter, Pat Taylor, Ruwan Parakramawansha, Jack Perry, Gordon Blair, Allan MacRaild, Adrian Parry-Jones, Mary Johnes, Stephanie Lee, Kelly Marie Shaw, Ilse Burger, Martin Punter, Andrea Ingham, Jane Perez, Zin Naing, Jordi Morell, Tracy Marsden, Andrea Hall, Sally Marshall, Louise Harrison, Rowilson Jarapa, Edith Wood, Victoria O'Loughlin, David Cohen, Silvie Davies, Kelechi Njoku, Mushiya Mpelembue, Laura Burgess, Radim Licenik, Mmua Ngwako, Nabeela Nisar, Rangah Niranchanan, Tatjana Roganova, Rajaram Bathula, Joseph Devine, Anette David, Anne Oshodi, Fenglin Guo, Emmanuelle Owoyele, Varthi Sukdeo, Robert Ballantine, Mudhar Abbdul-saheb, Angela Chamberlain, Aberami Chandrakumar, Philip Poku, Kirsty Harkness, Catrin Blank, Emma Richards, Ali Ali, Faith Kibutu, Olesia Balitska, Kathryn Birchall, Pauline Bayliss, Clare Doyle, Kathy Stocks, Arshad Majis, Jo Howe, Christine Kamara, Luke Barron, Ahmad Maatouk, Ralf Lindert, Katy Dakin, Jessica Redgrave, Biju Bhaskaran, Isam Salih, Debs Kelly, Susan Szabo, Dawn Tomlin, Helen Bearne, Jean Buxton, Pauline Fitzell, Georgina Ayres, Afaq Saulat, Kathleen Horan, Joanne Garfield-Smith, Harbens Bhakri, Paul Guyler, Devesh Sinha, Thayalini Loganathan, Amber Siddiqui, Anwer Siddiqui, Lucy Coward, Swapna Kunhunny, Sharon Tysoe, Rajalakshmi Orath Prabakaran, Shyam Kelavkar, Sindhu Rashmi, David Ngo, Kheng Xiong Ng, Nisha Menon, Sweni Shah, Mark Barber, Derek Esson, Fiona Brodie, Talat Anjum, Mushtaq Wani, Manju Krishnan, Leanne Quinn, Jayne Spencer, Terry Jones, Helen Thompson-Jones, Lynne Dacey, Srikanth Chenna, Sharon Storton, Sarah Thomas, Teresa Beaty, Shelley Treadwell, Caroline Davies, Susan Tucker, Lynda Connor, Peter Slade, Glyn Gainard, Girish Muddegowda, Ranjan Sanyal, Alda Remegoso, Nenette Abano, Chelsea Causley, Racquel Carpio, Stephanie Stevens, Adrian Butler, Resti Varquez, Hayley Denic, Francis Alipio, Andrew Moores, Adrian Barry, Holly Maguire, Jeanette Grocott, Kay Finney, Sue Lyjko, Christine Roffe, Joanne Hiden, Phillip Ferdinand, Vera Cvoro, Khalil Ullah, Nicola Chapman, Mandy Couser, Susan Pound, Katrina McCormick, Sean Mcauley, Senthil Raghunathan, Faye Shelton, Amanda Hedstrom, Margi Godfrey, Diane Havard, Amanda Buck, Kailash Krishnan, Nicola Gilzeane, Jack Roffe, Judith Clarke, Katherine Whittamore, Saima Sheikh, Rekha Keshvara, Carla Jordan, Benjamin Jackson, Gwendoline Wilkes, Jason Appleton, Zhe Law, Oliver Matias, Evangelos Vasileiadis, Cathy Mason, Anthea Parry, Geraldine Landers, Melinda Holden, Basaam Aweid, Khalid Rashed, Linda Balian, Carinna Vickers, Elizabeth Keeling, Sarah Board, Joanna Allison, Clare Buckley, Barbara Williams-Yesson, Joanne Board, Tressy Pitt-Kerby, Alfonso Tanate, Diane Wood, Manohar Kini, Dinesh Chadha, Deborah Walstow, Rosanna Fong, Robert Luder, Tolu Adesina, Jill Gallagher, Hayley Bridger, Elodie Murali, Maneesh Bhargava, Chloe van Someren, Frances Harrington, Abhijit Mate, Ali James, Gillian Courtauld, Christine Schofield, Katja Adie, Linda Lucas, Kirsty Bond, Bev Maund, Sam Ellis, Paul Mudd, Martin James, Samantha Keenan, Angela Bowring, Julie Cageao, Hayley Kingwell, Caroline Roughan, Anthony Hemsley, Jane Sword, David Strain, Keniesha Miller, Anita Goff, Karin Gupwell, Kevin Thorpe, Hedley Emsley, Shuja Punekar, Alison McLoughlin, Sulaiman Sultan, Bindu Gregory, Sonia Raj, Donna Doyle, Keith Muir, Wilma Smith, Angela Welch, Fiona Moreton, Bharath Kumar Cheripelli, Salwa El Tawil, Dheeraj Kalladka, Xuya Huang, Nicola Day, Sankaranarayanan Ramachandran, Caroline Crosbie, Jennifer Elliot, Tony Rudd, Katherine Marks, Ajay Bhalla, Jonathan Birns, Sagal Kullane, Nic Weir, Christopher Allen, Vanessa Pressly, Pam Crawford, Emma Battersby-Wood, Alex Blades, Shuna Egerton, Ashleigh Walters, Sue Evans, James Richard Marigold, Fiona Smith, Gabriella Howard, Imogen Gartrell, Simon Smith, Robyn Creeden, Chloe Cox, Cherish Boxall, Jonathan Hewitt, Claire Nott, Procter Sarah, Jessica Whiteman, Steve Buckle, Rebecca Wallace, Rina Mardania, Jane Gray, Claire Triscott, Anand Nair, Jill Greig, Pratap Rana, Matthew Robinson, Mohammad Irfan Alam, Duncan Wilson, Caroline Watchurst, Maria Brezitski, Luci Crook, Ifan Jones, Azra Banaras, Krishna Patel, Renuka Erande, Caroline Hogan, Isabel Hostettler, Amy Ashton, Shez Feerick, Nina Francia, Nnebuife Oji, Emma Elliott, Talal Al-Mayhani, Christine Lerpiniere, Ruth Fraser, Dipankar Dutta, Pauline Brown, Deborah Ward, Fiona Davis, Jennifer Turfrey, Chloe Hughes, Kayleigh Collins, Rehana Bakawala, Susan O'Connell, Jon Glass, David Broughton, Dinesh Tryambake, Lynn Dixon, Kath Chapman, Andrew Young, Adrian Bergin, Andrew Sigsworth, Aravind Manoj, Glyn Fletcher, Paula Lopez, Penelope Cox, Mark Wilkinson, Paul Fitzsimmons, Nikhil Sharma, James Choulerton, Denise Button, Lindsey Dow, Lukuman Gbadamoshi, Joanne Avis, Barbara Madigan, Stephanie McCann, Louise Shaw, Deborah Howcroft, Suzanne Lucas, Andrew Stone, Gillian Cluckie, Caroline Lovelock, Brian Clarke, Neha Chopra, Natasha Clarke, Bhavini Patel, Kate Kennedy, Rebecca Williams, Adrian Blight, Joanna O'Reilly, Chukwuka Orefo, Nilofer Dayal, Rita Ghatala, Temi Adedoyin, Fran Watson, Sarah Trippier, Lillian Choy, Barry Moynihan, Usman Khan, Val Jones, Naomi Jeyaraj, Lourda Kerin, Kamy Thavanesan, Divya Tiwari, Chantel Cox, Anja Ljubez, Laura Tucker, Arshi Iqbal, Caroline Bagnall, Marketa Keltos, Josh Roberts, Becky Jupp, Catherine Ovington, Emily Rogers, Owen David, Jo Bell, Barbara Longland, Gail Hann, Martin Cooper, Mohammad Nasar, Anoja Rajapakse, Inez Wynter, Ijaz Anwar, Helen Skinner, Tarn Nozedar, Damian McArdle, Balakrishna Kumar, Susan Crawford, Arunkumar Annamalai, Alex Ramshaw, Clare Holmes, Sarah Caine, Mairead Osborn, Emily Dodd, Peter Murphy, Nicola Devitt, Pauline Baker, Amy Steele, Lucy Belle Guthrie, Samantha Clarke, Ahamad Hassan, Dean Waugh, Emelda Veraque, Linetty Makawa, Mary Kambafwile, Marc Randall, Vasileios Papavasileiou, Claire Cullen, Jenny Peters, Hlaing Thant, Tanya Ingram, Mellor Zoe, Ramesh Durairaj, Melanie Harrison, Sarah Stevenson, Daniela Shackcloth, Jordan Ewing, Victoria Sutton, Mark McCarron, Jacqueline McKee, Mandy Doherty, Ferghal McVerry, Caroline Blair, Mary MacLeod, Janice Irvine, Heather Gow, Jacqueline Furnace, Anu Joyson, Baljit Jagpal, Sarah Ross, Katrina Klaasen, Sandra Nelson, Rebecca Clarke, Nichola Crouch, Beverly MacLennan, Vicky Taylor, Daniel Epstein, Ifan Jones, Avani Shukla, Vinodh Krishnamurthy, Paul Nicholas, Sammie Qureshi, Adam Webber, Justin Penge, Hawraman Ramadan, Stuart Maguire, Chris Patterson, Ruth Bellfield, Brigid Hairsine, Kelvin Stewart, Michaela Hooley, Outi Quinn, Bella Richard, Sally Moseley, Claire Nott, Steve Buckle, Procter Sarah, Jessica Whiteman, Mandy Edwards, Heidi Lawson, Rebecca Wallace, Claire Triscott, Michelle Tayler, Yogish Pai, Mahesh Dhakal, Bernard Esisi, Sofia Dima, Gemma Marie Smith, Mark Garside, Muhammad Naeem, Vidya Baliga, Gill Rogers, Ellen Brown, David Bruce, Rachel Hayman, Susan Clayton, Ed Gamble, Rebecca Grue, Bethan Charles, Adam Hague, Sujata Blane, Caroline Lambert, Afnan Chaudhry, Thomas Harrison, Kari Saastamoinen, Dionne Hove, Laura Howaniec, Gemma Grimwood, Ozlem Redjep, Fiona Humphries, Lucia Argandona, Larissa Cuenoud, Esther Erumere, Sageet Amlani, Grace Auld, Afraim Salek-Haddadi, Ursula Schulz, James Kennedy, Gary Ford, Philip Mathieson, Ian Reckless, Rachel Teal, Giulia Lenti, George Harston, Eoin O'Brien, Joanne Mcgee, Jennifer Mitchell, Elaine Amis, Dominic Handley, Siobhan Kelly, George Zachariah, Jobbin Francis, Sarah Crisp, Juliana Sesay, Sarah Finlay, Helen Hayhoe, Niamh Hannon, Tom Hughes, Bethan Morse, Henry De Berker, Emma Tallantyre, Ahmed Osman, Susan White, Stefan Schwarz, Benjamin Jelley, Rajendra Yadava, Khalid Azhar, Julie Reddan, Mirriam Sangombe, Samantha Stafford, Ken Fotherby, Debbie Morgan, Farrukh Baig, Karla Jennings-Preece, Donna Butler, Nasar Ahmad, Angela Willberry, Angela Stevens, Baljinder Rai, Prasad Siddegowda, Peter Howard, Afaq Saulat, Lisa Hyatt, Tracey Dobson, David Jarrett, Suheil Ponnambath, Jane Tandy, Yasmin Harrington-Davies, Rebecca Butler, Claire James, Stacey Valentine, Anne Suttling, Peter Langhorne, Gillian Kerr, Fiona Wright, Ruth Graham, Christine McAlpine, Mohammad Shahzad Iqbal, Louise Humphreys, Kath Pasco, Olga Balazikova, Ashraf Nasim, Cassilda Peixoto, Louise Gallagher, Shahrzad Shahmehri, Sandip Ghosh, Elizabeth Barrie, Danielle Gilmour, Margo Henry, Tom Webb, Linda Cowie, Hannah Rudenko, Shanni McDonald, Natasha Schumacher, Susannah Walker, Tracey Cosier, Anna Verrion, Eva Beranova, Audrey Thomson, Marius Venter, Arindam Kar, Sheila Mashate, Kirsten Harvey, Léjeune Gardener, Vinh Nguyen, Omid Halse, Olivia Geraghty, Beth Hazel, Peter Wilding, Victoria Tilley, Bernard Esisi, Tim Cassidy, Beverley McClelland, Maria Bokhari, Timothy England, Amanda Hedstrom, Mohana Maddula, Richard Donnelly, Paul Findlay, Ashish Macaden, Ian Shread, Charlotte Barr, Azlisham Mohd Nor, Claire Brown, Nicola Persad, Charlotte Eglinton, Marie Weinling, Benjamin Hyams, Alex Shah, John Baker, Anthony Byrne, Caroline McGhee, Amanda Smart, Claire Copeland, Michael Carpenter, Marion Walker, Richard Davey, Ann Needle, Razik Fathima, Gavin Bateman, Prabal Datta, Andrew Stanners, Linda Jackson, Julie Ball, Michelle Davis, Natalie Atkinson, Michelle Fawcett, Teresa Thompson, Helen Guy, Valerie Hogg, Carole Hays, Stephen Woodward, Mohammad Haque, Eluzai Hakim, Stuart Symonds, Mehran Maanoosi, Jane Herman, Toby Black, Skelton Miriam, Caroline Clarke, Alpha Anthony, Michele Tribbeck, Julie Cronin, Denise Mead, Ruth Fennelly, James McIlmoyle, Christina Dickinson, Carol Jeffs, Sajjad Anwar, Joanne Howard, Kirsty Jones, Saikat Dhar, Caroline Clay, Muhammad Siddiq, Simone Ivatts, Yolanda Baird, Moore Sally, Isobel Amey, Sophie Newton, Lisa Clayton-Evans, Indra Chadbourn, Rayessa Rayessa, Charde Naylor, Alicia Rodgers, Lisa Wilson, Sarah Wilson, Emma Clarkson, Ruth Davies, Paula Owings, Graeme Sangster, Valerie Gott, Victoria Little, Pauline Weir, Suja Cherian, Deepa Jose, Helen Moroney, Susan Downham, Angela Dodd, Venetia Vettimootal Johnson, Laura Codd, Naomi Robinson, Ashraf Ahmed, Mo Albazzaz, Sharon Johnson, Carol Denniss, Mishell Cunningham, Tajammal Zahoor, Timothy Webster, Sandra Leason, Syed Haider, Kausic Chatterjee, Arumugam Nallasivan, Charlotte Perkins, Samantha Seagrave, Colin Jenkins, Fiona Price, Claire Hughes, Lily Mercer, Malik Hussain, Sarah Brown, Miriam Harvey, Jane Homan, Mohammad Khan, Robert Whiting, Leanne Foote, Nicholas Hunt, Helen Durman, Lucy Brotherton, Jayne Foot, Corinne Pawley, Eliza Foster, Alison Whitcher, Kneale Metcalf, Jenny Jagger, Susan McDonald, Kelly Waterfield, Patrick Sutton, Naval Shinh, Ajmal Anversha, Garth Ravenhill, Richard Greenwood, Janak Saada, Alison Wiltshire, Rebekah Perfitt, Sreeman Andole, Naveen Gadapa, Karen Dunne, Magdalini Krommyda, Evelyne Burssens, Sam King, Catherine Plewa, Nigel Smyth, Jenny Wilson, Elio Giallombardo, Charlotte Eglinton, Lucy Sykes, Pradeep Kumar, James Barker, Isabel Huggett, Linda Dunn, Charlotte Culmsee, Philip Thomas, Min Myint, Richard O'Brien, Helen Brew, Nikhil Majmudar, Janice O'Connell, George Bunea, Charlotte Fox, Diane Gulliver, Andrew Smith, Betty Mokoena, Naweed Sattar, Ramesh Krishnamurthy, Emily Osborne, David Wilson, Belinda Wroath, Kevin Dynan, Michael Power, Susan Thompson, Victoria Adell, Enoch Orugun, Una Poultney, Rachel Glover, Hannah Crowther, Sarah Thornthwaite, Ivan Wiggam, Aine Wallace, Enda Kerr, Ailsa Fulton, Annemarie Hunter, Suzanne Tauro, Sarah Cuddy, David Mangion, Anne Hardwick, Skarlet Markova, Tara Lawrence, Carmen Constantin, Jo Fletcher, Isobel Thomas, Kerry Pettitt, Lakshmanan Sekaran, Margaret Tate, Kiranjit Bharaj, Rohan Simon, Frances Justin, Sakthivel Sethuraman, Duke Phiri, Niaz Mohammed, Meena Chauhan, Khaled Elfandi, Uzma Khan, Samantha Stafford, Julie Reddan, David Eveson, Amit Mistri, Lisa Manning, Shagufta Khan, Champa Patel, Mohammed Moqsith, Saira Sattar, Man Yee Lam, Kashif Musarrat, Claire Stephens, Latheef Kalathil, Richard Miller, Maqsud Salehin, Nikki Gautam, Duncan Bailey, Kelly Amor, Julie Meir, Anne Nicolson, Javed Imam, Lisa Wood, Julie White, Mahmud Sajid, George Ghaly, Margaret Ball, Rachel Gascoyne, Harald Proeschel, Simon Sharpe, Sarah Horton, Emily Beaves, Stephanie Jones, Brigitte Yip, Murdina Bell, Linda MacLiver, Brian MacInnes, Derek Esson, Don Sims, Jennifer Hurley, Mark Willmot, Claire Sutton, Edward Littleton, Susan Maiden, Rachael Jones, James Cunningham, Carole Green, Michelle Bates, Raj Shekhar, Kelly Waterfield, Ellie Gilham, Iman Ahmed, Rachel Crown, Tracy Fuller, Neetish Goorah, Angela Bell, Christine Kelly, Arun Singh, Jamie Walford, Benjamin Tomlinson, Farzana Patel, Stephen Duberley, Ingrid Kane, Chakravarthi Rajkumar, Jane Gaylard, Joanna Breeds, Nicola Gainsborough, Alexandra Pitt-Ford, Emma Barbon, Laura Latter, Philip Thompson, Simon Hervey, Shrivakumar Krishnamoorthy, Joseph Vassallo, Deborah Walter, Helen Cochrane, Meena Srinivasan, Robert Campbell, Denise Donaldson, Nichola Motherwell, Frances Hurford, Indranil Mukherjee, Antony Kenton, Sheila Nyabadza, Irene Martin, Benjamin Hunt, Hardi Hassan, Sarah O'Toole, Bander Dallol, Janet Putterill, Ratneshwari Jha, Rachel Gallifent, Puneet Kakar, Aparna Pusalkar, Kelly Chan, Puneet Dangri, Hannah Beadle, Angela Cook, Karen Crabtree, Santhosh Subramonian, Peter Owusu-Agyei, Natalie Temple, Nicola Butterworth-Cowin, Suzanne Ragab, Kerstin Knops, Emma Jinks, Christine Dickson, Laura Gleave, Judith Dube, Jacqui Leggett, Tatiana Garcia, Sissy Ispoglou, Rachel Evans, Sandeep Ankolekar, Anne Hayes, Hlaing Ni, Bithi Rahman, Josette Milligan, Carol Graham, Josin Jose, Breffni Keegan, Mandy Doherty, Jim Kelly, Caroline Blair, Richard Dewar, James White, Kelly Thomas

## Abstract

**Background:**

Antiplatelet therapy reduces the risk of major vascular events for people with occlusive vascular disease, although it might increase the risk of intracranial haemorrhage. Patients surviving the commonest subtype of intracranial haemorrhage, intracerebral haemorrhage, are at risk of both haemorrhagic and occlusive vascular events, but whether antiplatelet therapy can be used safely is unclear. We aimed to estimate the relative and absolute effects of antiplatelet therapy on recurrent intracerebral haemorrhage and whether this risk might exceed any reduction of occlusive vascular events.

**Methods:**

The REstart or STop Antithrombotics Randomised Trial (RESTART) was a prospective, randomised, open-label, blinded endpoint, parallel-group trial at 122 hospitals in the UK. We recruited adults (≥18 years) who were taking antithrombotic (antiplatelet or anticoagulant) therapy for the prevention of occlusive vascular disease when they developed intracerebral haemorrhage, discontinued antithrombotic therapy, and survived for 24 h. Computerised randomisation incorporating minimisation allocated participants (1:1) to start or avoid antiplatelet therapy. We followed participants for the primary outcome (recurrent symptomatic intracerebral haemorrhage) for up to 5 years. We analysed data from all randomised participants using Cox proportional hazards regression, adjusted for minimisation covariates. This trial is registered with ISRCTN (number ISRCTN71907627).

**Findings:**

Between May 22, 2013, and May 31, 2018, 537 participants were recruited a median of 76 days (IQR 29–146) after intracerebral haemorrhage onset: 268 were assigned to start and 269 (one withdrew) to avoid antiplatelet therapy. Participants were followed for a median of 2·0 years (IQR [1·0– 3·0]; completeness 99·3%). 12 (4%) of 268 participants allocated to antiplatelet therapy had recurrence of intracerebral haemorrhage compared with 23 (9%) of 268 participants allocated to avoid antiplatelet therapy (adjusted hazard ratio 0·51 [95% CI 0·25–1·03]; p=0·060). 18 (7%) participants allocated to antiplatelet therapy experienced major haemorrhagic events compared with 25 (9%) participants allocated to avoid antiplatelet therapy (0·71 [0·39–1·30]; p=0·27), and 39 [15%] participants allocated to antiplatelet therapy had major occlusive vascular events compared with 38 [14%] allocated to avoid antiplatelet therapy (1·02 [0·65–1·60]; p=0·92).

**Interpretation:**

These results exclude all but a very modest increase in the risk of recurrent intracerebral haemorrhage with antiplatelet therapy for patients on antithrombotic therapy for the prevention of occlusive vascular disease when they developed intracerebral haemorrhage. The risk of recurrent intracerebral haemorrhage is probably too small to exceed the established benefits of antiplatelet therapy for secondary prevention.

**Funding:**

British Heart Foundation.

## Introduction

Adults with stroke due to spontaneous intracerebral haemorrhage often have a history of occlusive vascular disease, such as myocardial infarction or ischaemic stroke.^1^ Consequently, at least a third of adults in high-income countries are taking oral antithrombotic (antiplatelet or anticoagulant) drugs at the onset of intracerebral haemorrhage.^2^ Generally, antithrombotic drugs are immediately discontinued because of the risk of early haematoma growth. Discontinuation of these drugs is often permanent because of the perceived risk of recurrent intracerebral haemorrhage. However, the risk of occlusive vascular events might be higher,^3^ so resumption of antithrombotic therapy might be beneficial overall.

Results of randomised trials have found a favourable balance of the benefits and risks of antiplatelet and anticoagulant therapy for the secondary prevention of occlusive vascular disease for a variety of conditions, but these trials excluded people with a history of major bleeding.^4^, ^5^, ^6^ Therefore, no published randomised trials are available on whether long-term antithrombotic therapy is safe or beneficial for survivors of intracerebral haemorrhage overall,^7^ or in subgroups who are at higher risk of bleeding, such as people with lobar intracerebral haemorrhage.^1^

The use of antiplatelet therapy for about 2 days did not result in adverse effects for patients who had been enrolled in randomised trials of aspirin, without knowing their stroke was due to intracerebral haemorrhage.^8^ In the longer term (months to years), findings from a systematic review and meta-analysis^9^ of observational studies of patients with any type of intracranial haemorrhage (ie, intracerebral, subarachnoid, or subdural haemorrhage) showed lower risks of occlusive vascular events and no difference in haemorrhagic events associated with resumption compared with avoidance of antiplatelet therapy. Small, non-randomised observational studies of patients with intracerebral haemorrhage have reported similar associations with starting antiplatelet therapy compared with its avoidance.^10^, ^11^, ^12^, ^13^, ^14^ Because of the paucity of evidence, no guidelines with strong recommendations about long-term antiplatelet therapy after intracerebral haemorrhage are available,^15^, ^16^ so variations in clinical practice occur.^3^ Therefore, randomised controlled trials are needed to establish whether to use antiplatelet therapy after intracerebral haemorrhage.^7^

**Research in context**

**Evidence before this study**

The Antithrombotic Trialists' Collaboration meta-analysis of randomised controlled trials found that aspirin use for the secondary prevention of occlusive vascular disease reduces risk of major vascular events, even though it might increase the risk of intracranial haemorrhage (a composite of intracerebral, subarachnoid, or subdural haemorrhages). However, these trials excluded patients with intracerebral haemorrhage, the commonest subtype of intracranial haemorrhage with the worst outcome. We searched the Cochrane Stroke Group Register, the Cochrane Central Register of Controlled Trials, Ovid MEDLINE (from 1948), Ovid Embase (from 1980), online registries of clinical trials, and bibliographies of relevant publications on Jan 28, 2019, (appendix) for randomised controlled trials of starting versus avoiding antiplatelet therapy after intracerebral haemorrhage, from database inception until Jan 28, 2019, without language restrictions. We found no completed randomised controlled trials. A meta-analysis of observational studies found no difference in the risk of haemorrhagic events and a lower risk of occlusive vascular events associated with antiplatelet therapy resumption after any type of intracranial haemorrhage.

**Added value of this study**

The REstart or STop Antithrombotics Randomised Trial (RESTART) is the first randomised controlled trial comparing the effects of starting versus avoiding antiplatelet therapy for patients with previous intracerebral haemorrhage that occurred while taking antithrombotic (antiplatelet or anticoagulant) therapy. Participants allocated to start antiplatelet therapy experienced proportionally (but not statistically) fewer recurrences of intracerebral haemorrhage (adjusted hazard ratio 0·51 [95% CI 0·25–1·03]; p=0·060), fewer major haemorrhagic events (0·71 [0·39–1·30]; p=0·27), and similar numbers of major occlusive vascular events (1·02 [0·65–1·60]; p=0·92), compared with participants allocated to avoid antiplatelet therapy. These results exclude all but a very modest increase in the risk of recurrent intracerebral haemorrhage with antiplatelet therapy. The risk of recurrent intracerebral haemorrhage is probably too small to exceed the established benefits of antiplatelet therapy for the secondary prevention of occlusive vascular disease.

**Implications of all the available evidence**

RESTART's findings provide reassurance about the safety of antiplatelet therapy after intracerebral haemorrhage that occurred while taking antithrombotic therapy. Replication of these findings and investigation of the possibility that antiplatelet therapy reduces the risk of recurrent intracerebral haemorrhage require further investigation in ongoing randomised trials (RESTART-Fr NCT02966119 and STATICH NCT03186729), a subsequent meta-analysis of RESTART, and an adequately powered definitive randomised controlled trial.

We initiated the REstart or STop Antithrombotics Randomised Trial (RESTART) with the aim of estimating the relative and absolute effects of starting versus avoiding antiplatelet therapy on recurrent symptomatic intracerebral haemorrhage and whether this risk might exceed any reduction of occlusive vascular events.^17^.

## Methods

### Study design

RESTART was an investigator-led, pragmatic, multicentre, prospective, randomised, open-label, blinded endpoint, parallel-group trial in 122 hospitals in the UK. The Scotland A Research Ethics Committee approved the trial protocol (Nov 2, 2012).^17^ The trial co-sponsors were the University of Edinburgh and National Health Service Lothian Health Board. The patient reference group for the Research to Understand Stroke due to Haemorrhage (RUSH) programme reviewed the study materials and progress. The trial steering committee and co-sponsors approved the trial protocol and the statistical analysis plan.^17^, ^18^

### Participants

We included adults (≥18 years) who had survived at least 24 h after spontaneous intracerebral haemorrhage confirmed by brain imaging and were taking antithrombotic (antiplatelet or anticoagulant) therapy for the prevention of occlusive vascular disease at the onset of intracerebral haemorrhage, after which therapy was discontinued. Patients were ineligible if the intracerebral haemorrhage was attributable to preceding head injury, haemorrhagic transformation of an ischaemic stroke, or intracranial haemorrhage without intracerebral haemorrhage; if they were still taking antithrombotic therapy at the time of consent (ie, after intracerebral haemorrhage); if they were pregnant, breastfeeding, or of childbearing age and not taking contraception; or if they or their carer was unable to understand spoken or written English. Patients, or their nearest relative or representative if the patient did not have mental capacity, provided written informed consent in inpatient or outpatient hospital settings. Participants could be enrolled if they or their nearest relative, and their physician in secondary care, were uncertain about whether to start or avoid antiplatelet therapy and had consented, in which case randomisation was done at least 24 h after stroke symptom onset.^17^

### Randomisation and masking

Investigators supplied complete information about participants' demographics, comorbidities, functional status, previous antithrombotic therapy, intracerebral haemorrhage, and their preferred antiplatelet therapy into a database via a secure web interface with in-built validation to ensure complete baseline data before randomisation. A central computerised randomisation system incorporating a minimisation algorithm randomly assigned participants (1:1) to start or avoid antiplatelet therapy. The algorithm randomly allocated the first participant with a probability of 0·5 to one group of the trial. Thereafter, adaptive stratification (ie, minimisation) allocated each subsequent participant with a probability of 0·8 to the group that minimised differences between the two groups of the trial with respect to five baseline variables: qualifying intracerebral haemorrhage location (lobar *vs* non-lobar); time since symptom onset (1–6 days, 7–30 days, >30 days); antiplatelet therapy preferred by the patient's physician if allocated to start (aspirin alone *vs* other antiplatelet therapy); participant age at randomisation (<70 years *vs* ≥70 years); and predicted probability of being alive and independent at 6 months (<0·15 *vs* ≥0·15).^19^ The five variables were weighted equally, and the weights were constant over the duration of recruitment. The web interface displayed each participant's unique study identification number and their allocation to either starting or avoiding antiplatelet therapy, which was also sent in an email to all investigators at the hospital site, having been concealed until that point. If the participant was allocated to start antiplatelet therapy, the system reminded investigators to prescribe the prespecified preferred antiplatelet therapy within 24 h.

Treatment allocation was open to participants, the clinicians caring for them in primary and secondary care, and local investigators. Staff following up the participants at the trial coordinating centre were masked to treatment allocation. Outcome event adjudicators were masked to participant identity, treatment allocation, and drug use.

### Procedures

Participants who had not already been imaged with MRI but complied with the trial's MRI protocol, and who were able and willing to undergo brain MRI, provided informed consent and had a brain MRI scan before randomisation. After randomisation, anyone of a panel of consultant neuroradiologists (PMW, DPM, DM, PB, JCduP, or YJ), who was masked to treatment allocation, used the web-based Systematic Image Review System tool to review anonymised DICOM images of diagnostic brain CT or MRI to confirm or refute eligibility and to support the adjudication of cerebral outcome events.

The intervention of starting antiplatelet therapy was restricted to the use of one or more of oral aspirin, dipyridamole, or clopidogrel, begun within 24 h of randomisation with doses determined at the discretion of the consultant responsible for the participant. The comparator was a policy of avoiding antiplatelet therapy (ie, no placebo group). Participants were permitted to start or discontinue antiplatelet or anticoagulant therapy if clinically indicated by events during follow-up, regardless of treatment allocation. We measured adherence after randomisation regardless of treatment allocation by recording antiplatelet therapy use before the first outcome event according to the preceding clinic or hospital discharge form or follow-up questionnaire. We collected information about blood-pressure lowering drugs and blood pressure control at discharge and during follow-up.

We followed up participants by sending a postal questionnaire to their primary care practitioners (who hold a comprehensive lifelong medical record for each patient registered with them), followed by a postal questionnaire to surviving participants (or carers) who had not withdrawn, to check vital status, medication use, modified Rankin scale score, and the occurrence of outcomes. We sent questionnaires at set intervals after randomisation (6 months or 1 year, 2 years, 3 years, 4 years, and 5 years). Participants who did not respond to the questionnaire were interviewed by phone.^20^, ^21^

We recorded serious adverse events (that were neither an outcome event nor an expected complication of stroke) via investigators if they occurred before hospital discharge or via primary care practitioners' annual reports of hospital admissions. Investigators reported protocol deviations and violations to the trial coordinating centre and the sponsor.

Monitoring included central statistical monitoring of trial conduct, data quality, and participant safety, supplemented by triggered onsite monitoring visits if required and detailed source data verification at the trial coordinating centre. All baseline and outcome data underwent completeness, range, consistency, validation, and logic checks within the web-based case report forms.

### Outcomes

The primary outcome was fatal or non-fatal radiographically or pathologically proven recurrent symptomatic intracerebral haemorrhage assessed in all participants (except one participant who withdrew before the first follow-up).^17^ The secondary outcomes were a composite of all major haemorrhagic events and a composite of all major occlusive vascular events.^17^ Major haemorrhagic events included recurrent symptomatic intracerebral haemorrhage (primary outcome), other forms of symptomatic spontaneous or traumatic intracranial haemorrhage, and symptomatic major extracranial haemorrhage at any site (requiring transfusion or endoscopic treatment or surgery, or resulting in death within 30 days).^18^ Major occlusive vascular events were ischaemic stroke; myocardial infarction; mesenteric ischaemia; peripheral arterial occlusion; deep vein thrombosis; pulmonary embolism; and carotid, coronary, or peripheral arterial revascularisation procedures.^18^ The composite secondary outcome of all major haemorrhagic or occlusive vascular events combined these two composite outcomes.^18^ In the protocol, we had specified a composite secondary outcome of all major vascular events defined by the Antithrombotic Trialists' Collaboration (non-fatal myocardial infarction, non-fatal stroke [ischaemic, haemorrhagic, or uncertain cause], or death from a vascular cause).^4^, ^17^

Two consultant neurologists (WNW and MRM) at the trial coordinating centre were internal assessors of reports of every outcome event, masked to treatment allocation and use of antithrombotic therapy, using all available source documentation including clinical records, death certificates, autopsy reports, imaging reports, outpatient clinic letters, and hospital discharge summaries. One consultant neurologist (TG) was an external assessor and reviewed the same information on a random sample of 25 internally assessed events. He agreed with the internal assessors for 24 (96%) events (appendix) and, therefore, the internal assessors' categorisations remained final. Standardised definitions guided the final categorisation of outcomes.^22^, ^23^

### Statistical analysis

We aimed to recruit 720 participants and follow them up for at least 2 years to cover several combinations of published estimates of the primary outcome event rate in cohort studies (1·8–7·4% per year)^1^ and an up to four-times proportional increase in the absolute risk of the primary outcome with the use of antiplatelet therapy in observational studies.^10^, ^11^, ^12^ For example, at the 5% significance level, the trial would have 90% power to detect a doubling of a primary outcome event rate of 4·5% per year, or 93% power to detect a four-times increase of a rate of 1% per year.^17^

Throughout the recruitment period, unmasked trial statisticians supplied the independent data monitoring committee with analyses of the accumulating baseline and follow-up data in strict confidence at least once every year, so that they could assess trial conduct, safety, and efficacy, and make recommendations to the trial steering committee. No formal fixed schedule of interim analyses was followed, but the data monitoring committee could advise the chairman of the trial steering committee if they thought the randomised comparisons provided proof beyond reasonable doubt that, for some patients, antiplatelet therapy was clearly indicated or contraindicated in clinical practice.

Without reference to data by randomised allocation or input from the unmasked trial statistician, the masked trial statistician (GDM) and chief investigator (RA-SS) prepared a statistical analysis plan that was approved by the trial steering committee before database lock, and then published.^18^

We estimated the survival function in each treatment group using a Kaplan-Meier survival analysis of time to first occurrence of a primary or secondary outcome event during all available follow-up after randomisation, censored at death unrelated to an outcome event or last available follow-up. We quantified completeness of follow-up as the proportion of participants with a complete follow-up questionnaire at each planned interval after randomisation, and as the proportion of the planned duration of follow-up that was observed.^24^ We quantified absolute differences in annual event rates. After assessing the proportional hazards assumption graphically and including a treatment by log(time) interaction, we compared the survival functions by allocated treatment using the log-rank test. We constructed an unadjusted Cox proportional hazards regression model and a second model adjusted for all five covariates included in the minimisation algorithm (which was the primary method of analysis) to calculate the hazard ratios (HRs). We prespecified a hierarchical testing of the primary outcome, key secondary outcomes, and other secondary outcomes, so we did not adjust the threshold of statistical significance for multiplicity.^18^ We used the Mann–Whitney test to compare group summaries of modified Rankin scale scores by randomised group. We did sensitivity analyses by adding symptomatic stroke of uncertain subtype or deaths of undetermined cause to the primary outcome, and by calculating the cumulative incidence of all major haemorrhagic or occlusive vascular events.

We did prespecified exploratory subgroup analyses of the primary and secondary outcomes with statistical tests of interaction to estimate heterogeneity of treatment effect between the prespecified subgroups: the five covariates used by the minimisation algorithm, antithrombotic therapy before intracerebral haemorrhage, and history of atrial fibrillation.

The unmasked trial statistician (JS) did all statistical analyses with SAS version 9.4.

This trial is registered with ISRCTN (number ISRCTN71907627).

### Role of the funding source

The funder of this study had no role in study design, data collection, data analysis, data interpretation, or writing and decision to publish this Article. The corresponding author had full access to all data in the trial and had final responsibility for the decision to submit for publication.

## Results

**Figure 1:**
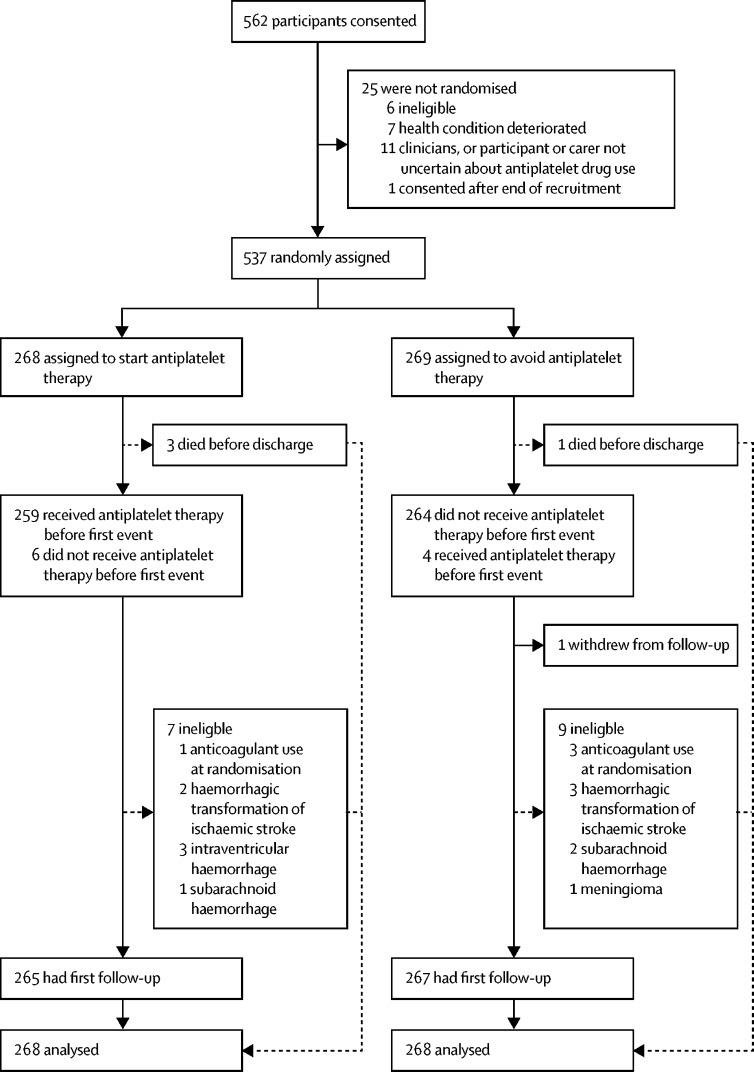
Trial profile

Between May 22, 2013, and May 31, 2018, 562 participants consented to participate in the study, from 104 of 122 activated hospital sites (appendix, figure 1). 20 participants also enrolled in RESTART after they enrolled in the Tranexamic acid for hyperacute primary IntraCerebral Haemorrhage (TICH-2) trial. Although the planned period of recruitment was extended by 1 year (until May 31, 2018), we did not achieve the planned sample size after a time-limited extension agreed by the funder, because only one in 12 eligible patients was recruited;^25^ therefore, we increased the duration of follow-up by 1 year to accrue the planned numbers of person-years of follow-up and primary outcome events.

268 participants were randomly assigned to start antiplatelet therapy and 269 to avoid antiplatelet therapy (figure 1), of whom all but one participant in the avoidance group were included in the outcome analyses.

At baseline, participants in the two treatment groups were on average 76 years old, approximately two-thirds were male, and 92% were white (table 1). 62% of participants had lobar intracerebral haemorrhage, 88% had one or more previous occlusive vascular disease (mostly ischaemic heart disease, ischaemic stroke, or transient ischaemic attack), three-quarters had a history of high blood pressure, a quarter had diabetes, and a quarter had atrial fibrillation when they were randomised (appendix). Participants were randomly assigned to each group a median of 76 days (IQR 29–146) after intracerebral haemorrhage onset. Half of the participants were taking aspirin, about a quarter clopidogrel, and approximately one-fifth oral anticoagulation at the onset of intracerebral haemorrhage (appendix). At baseline, participants' characteristics and use of antithrombotic therapy were well balanced for major prognostic factors and potential confounders (table 1). With the exception of 12 participants who were ineligible because their intracranial haemorrhage did not extend into the brain parenchyma or had intracerebral haemorrhages that were found to be secondary to a macrovascular cause (one cavernous malformation, one venous thrombosis, and one aneurysm) and four who were taking anticoagulants at randomisation, the remaining 522 (97%) participants had intracerebral haemorrhage without an underlying structural or macrovascular cause identified.

**Table 1:** Baseline characteristics

			**Start antiplatelet therapy (n=268)**	**Avoid antiplatelet therapy (n=269)**
Sex				
	Male		173 (65%)	187 (70%)
	Female		95 (35%)	82 (30%)
Age*				
	Overall		77 (69–82)	76 (69–82)
	<70 years		73 (27%)	73 (27%)
	≥70 years		195 (73%)	196 (73%)
Ethnicity				
	White		251 (94%)	242 (90%)
	Asian		12 (4%)	18 (7%)
	Black		4 (1%)	5 (2%)
	Other		1 (<1%)	4 (1%)
Indication for antithrombotic therapy before intracerebral haemorrhage†				
	At least one occlusive vascular disease			
		With atrial fibrillation	42 (16%)	50 (19%)
		Without atrial fibrillation	194 (72%)	189 (70%)
	No occlusive vascular diseases			
		With atrial fibrillation	19 (7%)	23 (9%)
		Without atrial fibrillation	13 (5%)	7 (3%)
History of intracranial or extracranial haemorrhage†			22 (8%)	25 (9%)
Location of intracerebral haemorrhage*				
	Lobar supratentorial		166 (62%)	166 (62%)
	Non-lobar		102 (38%)	103 (38%)
Time since intracerebral haemorrhage symptom onset*				
	Overall		80 (30–149)	71 (29–144)
	1–6 days		10 (4%)	11 (4%)
	7–30 days		59 (22%)	59 (22%)
	>30 days		199 (74%)	199 (74%)
Probability of good 6-month outcome*^19^				
	<0·15		48 (18%)	51 (19%)
	≥0·15		220 (82%)	218 (81%)
Context of enrolment				
	Hospital inpatient		87 (32%)	96 (36%)
	Hospital outpatient		181 (68%)	173 (64%)
	Participant consented		212 (79%)	213 (79%)
	Proxy consented		56 (21%)	56 (21%)

Data are n (%) or median (IQR).

* Variables used in the minimisation algorithm.

† Complete list of comorbidities is in the appendix.

Follow-up ended on Nov 30, 2018. Four participants died (figure 1) before hospital discharge, and the remaining 533 participants were followed up at hospital or clinic discharge. Completeness of primary care practitioner questionnaires (79% by post, 16% by telephone, and 4% by both) was 100% at all follow-up timepoints (from 6 months to 4 years). Completeness of participant or carer questionnaires (60% by post, 38% by telephone, and 2% by both) was 99% at 6 months or at 1 year, 99% at 2 years, 98% at 3 years, and 94% at 4 years. We obtained 1064 of an intended 1071 person-years of follow-up (overall completeness 99·3%).

Immediate adherence to allocated treatment was good, with some decline over time: 99% at discharge, 93% after 6 months or 1 year, 89% after 2 years, 86% after 3 years, and 82% after 4 years (appendix). Few participants (≤10%) used anticoagulant therapy during follow-up (appendix). Most participants took at least one blood-pressure lowering drug during follow-up and achieved median systolic blood pressure 130 mm Hg, with good balance by treatment allocation (appendix).

The proportional hazards assumption was fulfilled for analyses of primary and secondary outcomes during follow-up.

**Figure 2:**
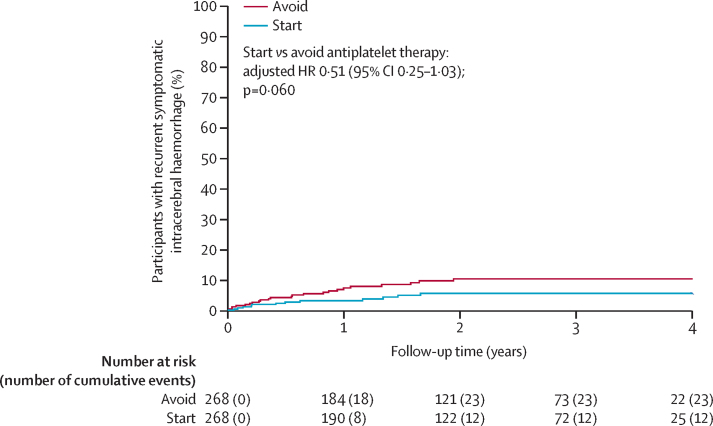
Kaplan-Meier plot of the first occurrence of recurrent symptomatic intracerebral haemorrhage Numbers at risk refer to survivors under follow-up at the start of each year according to treatment allocation. Cumulative events indicate the participants in follow-up with a first event. HR=hazard ratio.

**Figure 3:**
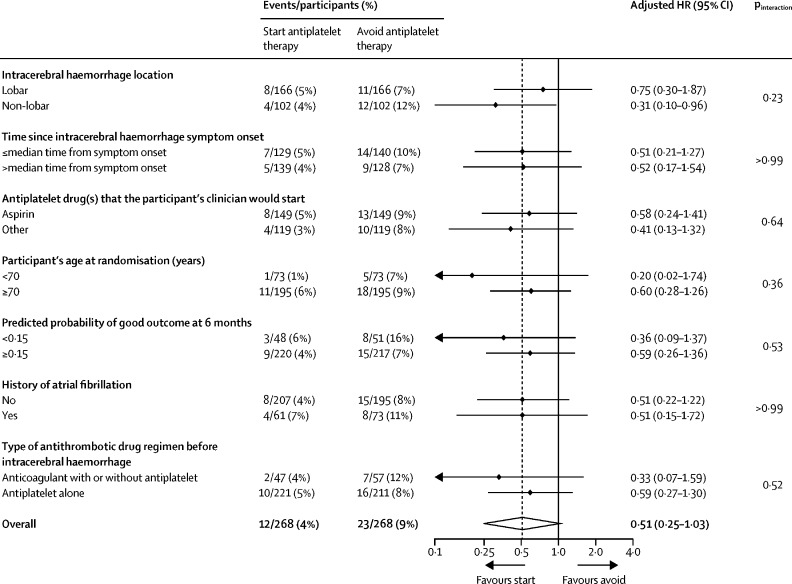
Prespecified exploratory subgroup analyses of the risk of first recurrent symptomatic intracerebral haemorrhage

For the primary outcome, 12 [4%] of 268 participants allocated to start antiplatelet therapy had recurrences of intracerebral haemorrhage compared with 23 [9%] of 268 participants who did not start therapy (adjusted HR 0·51 [95% CI 0·25–1·03]; p=0·060; Table 2, Table 3, figure 2, appendix). This proportional reduction in the primary outcome was similar in unadjusted and adjusted models, and in two sensitivity analyses involving the addition of symptomatic stroke of uncertain subtype (p=0·044) or death of undetermined cause (p=0·048) as possible occurrences of the primary outcome (table 3). 30-day case fatality after recurrent intracerebral haemorrhage was not different between participants starting antiplatelet therapy (5 [42%] of 12 participants) and those avoiding antiplatelet therapy (9 [39%] of 23 participants). No evidence was found of heterogeneity of the effects of antiplatelet therapy on the primary outcome in prespecified exploratory subgroup analyses (figure 3).

**Table 2:** Frequencies of the first occurrence and all primary and secondary outcome events during follow-up

			**Start antiplatelet therapy (n=268)**		**Avoid antiplatelet therapy (n=268)**	
			First event	All events	First event	All events
**Primary outcome**						
Recurrent symptomatic spontaneous intracerebral haemorrhage			12 (4%)	14	23 (9%)	27
**Secondary outcomes**						
Arterial events						
	Major haemorrhagic events					
		Spontaneous or traumatic intracranial extracerebral haemorrhage	4 (1%)	4	3 (1%)	3
		Major extracranial haemorrhage	4 (1%)	4	0	0
	Major occlusive vascular events					
		Ischaemic stroke	19 (7%)	21	27 (10%)	28
		Myocardial infarction	5 (2%)	5	8 (3%)	9
		Peripheral arterial occlusion	5 (2%)	5	2 (1%)	2
		Transient ischaemic attack	11 (4%)	12	18 (7%)	23
		Retinal arterial occlusion	0	0	0	0
		Mesenteric ischaemia	0	0	0	0
	Stroke of uncertain subtype		0	0	1 (<1%)	1
Carotid, coronary, or peripheral arterial revascularisation procedures			12 (4%)	12	5 (2%)	5
Venous events						
	Deep vein thrombosis		6 (2%)	6	2 (1%)	2
	Pulmonary embolism		4 (1%)	4	1 (<1%)	1
Deaths						
	Fatal outcome event		10 (4%)	10	19 (7%)	19
	Other cardiovascular death		6 (2%)	6	8 (3%)	8
	Sudden cardiac death		2 (1%)	2	0	0
	Non-cardiovascular death		35 (13%)	35	22 (8%)	22
	Undetermined cause		1 (<1%)	1	1 (<1%)	1

Data are n (%) or n.

**Table 3:** Risks of first occurrence of primary and secondary outcome events during follow-up

	**Start antiplatelet therapy (n=268)**	**Avoid antiplatelet therapy (n=268)**	**Log-rank test p value**	**Unadjusted analysis**		**Adjusted analysis**	
				HR (95% CI)	p value	HR (95% CI)	p value
**Primary outcome**							
Recurrent symptomatic spontaneous intracerebral haemorrhage	12	23	0·057	0·51 (0·26–1·03)	0·062	0·51 (0·25–1·03)	0·060
**Sensitivity analyses of the primary outcome**							
Recurrent symptomatic spontaneous intracerebral haemorrhage or symptomatic stroke of uncertain subtype	12	24	0·041	0·49 (0·25–0·99)	0·046	0·49 (0·24–0·98)	0·044
Recurrent symptomatic spontaneous intracerebral haemorrhage or death of undetermined cause	13	25	0·047	0·51 (0·26–1·00)	0·051	0·51 (0·26–0·99)	0·048
**Secondary outcomes**							
All major haemorrhagic events (all types of symptomatic spontaneous or traumatic intracranial haemorrhage, or symptomatic major extracranial haemorrhage)	18	25	0·27	0·71 (0·39–1·30)	0·27	0·71 (0·39–1·30)	0·27
All major occlusive vascular events (ischaemic stroke; myocardial infarction; mesenteric ischaemia; peripheral arterial occlusion; deep vein thrombosis; pulmonary embolism; or carotid, coronary, or peripheral arterial revascularisation procedures)	39	38	0·97	1·01 (0·65–1·58)	0·97	1·02 (0·65–1·60)	0·92
All major haemorrhagic or occlusive vascular events	54	61	0·42	0·86 (0·60–1·24)	0·42	0·86 (0·60–1·24)	0·43
Major occlusive vascular events*	45	52	0·39	0·84 (0·56–1·25)	0·39	0·84 (0·56–1·25)	0·39
Major vascular events (as defined by the Antithrombotic Trialists' Collaboration)	45	65	0·026	0·65 (0·45–0·95)	0·027	0·65 (0·44–0·95)	0·025

HR=hazard ratio.

* As defined in the trial protocol.

During follow-up (table 2), 104 (19%) participants died due to non-cardiovascular causes (n=57, 55%), primary or secondary outcome events (n=29, 28%), other cardiovascular deaths (n=16, 15%), or undetermined causes (n=2, 2%). 96 (18%) participants had at least one arterial major occlusive vascular event (including stroke of uncertain subtype), 46 (9%) had at least one major haemorrhagic event, 13 (2%) had at least one venous major occlusive vascular event, and 17 (3%) had a revascularisation procedure.

For the composite secondary outcomes,^18^ 18 (7%) of 268 participants allocated to start antiplatelet therapy experienced major haemorrhagic events compared with 25 (9%) of 268 participants allocated to avoid antiplatelet therapy (adjusted HR 0·71 [95% CI 0·39–1·30]; p=0·27); 39 (15%) of 268 participants in the antiplatelet group had major occlusive vascular events compared with 38 (14%) of 268 participants in the avoidance group (1·02 [0·65–1·60]; p=0·92); and 54 (20%) of 268 participants in the antiplatelet group had major haemorrhagic or occlusive vascular events compared with 61 (23%) of 268 participants in the avoidance group (0·86 [0·60–1·24]; p=0·43; table 3, appendix). In a sensitivity analysis, antiplatelet therapy did not reduce the cumulative incidence of all major haemorrhagic or occlusive vascular events (p=1·0). For the composite secondary outcome of all major vascular events specified in the trial protocol,^4^, ^17^ antiplatelet therapy seemed to reduce the risk of non-fatal myocardial infarction, non-fatal stroke (ischaemic, haemorrhagic, or uncertain cause), or death from a vascular cause (adjusted HR 0·65 [95% CI 0·44–0·95]; p=0·025; table 3, appendix). We found no evidence of heterogeneity of the effects of antiplatelet therapy on these secondary outcomes in prespecified exploratory subgroup analyses (appendix) or in the distribution of the modified Rankin scale score during follow-up (appendix). Few serious adverse events occurred (n=11), which were neither outcomes nor expected complications of stroke (appendix).

## Discussion

In this randomised trial of 537 survivors of an intracerebral haemorrhage while on antithrombotic therapy, starting antiplatelet therapy might have reduced the risk of recurrent symptomatic intracerebral haemorrhage. The results exclude all but a very modest increase in the risk of recurrent intracerebral haemorrhage with antiplatelet therapy, which seems too small to exceed the established benefits of antiplatelet therapy for secondary prevention.^4^ Therefore, starting antiplatelet therapy seems to be safe and might be beneficial in patients who survived a median of 76 days after intracerebral haemorrhage, most of whom had good functional ability at baseline and a higher probability of good functional outcome at 6-month follow up (table 1, appendix).^19^ Our findings, alongside published observational studies,^9^, ^10^, ^11^, ^12^, ^13^, ^14^ provide reassurance about the use of long-term antiplatelet therapy in a range of patients after intracerebral haemorrhage associated with antithrombotic therapy.

RESTART is the first randomised trial comparing starting versus avoiding antiplatelet therapy after intracerebral haemorrhage,^7^ and provides more reliable estimates of the effects of antiplatelet therapy than previous observational studies.^9^, ^10^, ^11^, ^12^, ^13^, ^14^ We minimised selection bias by using central, computerised random sequence generation and concealing allocation on the web application until all baseline data were entered. The recruited participants were comparable to European hospital-based and population-based cohorts of survivors of intracerebral haemorrhage who had previously taken antithrombotic therapy.^3^ Blood pressure was controlled for both groups throughout follow-up: half of the participants had a systolic blood pressure below the recommended target in the UK national stroke guideline and antihypertensive drug use was similar between groups. We minimised attrition bias by achieving 99·3% completeness of follow-up. The risk of recurrent intracerebral haemorrhage was at the lower end of the ranges reported in cohort studies,^1^ but similar to the 0·61–1·20% annual rates observed with cilostazol or aspirin use in the recent PICASSO trial.^26^ The risk of gastrointestinal haemorrhage was low (table 2), possibly due to the prevalence of proton pump inhibitor use in a contemporary population of older stroke survivors.^27^ We masked outcome assessors to treatment allocation group and receipt of antithrombotic therapy, and used objective definitions of major outcomes and independent verification, to reduce misclassification of haemorrhagic and occlusive vascular events, and reduce bias that can arise in outcome assessment when treatment allocation is open.^28^ We prespecified our outcomes and methods of analysis,^17^ and report these according to our protocol and statistical analysis plan.^18^ We also did prespecified exploratory analyses to investigate the effects of antiplatelet therapy according to brain imaging biomarkers that are often observed in clinical practice, which we report separately.^29^

RESTART has some limitations. We intended to recruit 720 participants and follow them up for 2 years, but we recruited only 537 people (75% of our target). Investigators managed to recruit only one in 12 eligible patients; the remainder were not randomised because 28% of patients were too unwell when approached, 26% of patients' physicians were certain about whether or not to use antiplatelet therapy, 9% of patients declined, 7% of patients were started on oral anticoagulation, and 30% were not recruited for other reasons.^25^ Because of slow recruitment, we did a stepped wedge cluster randomised study within this trial at 72 of the sites to investigate whether stroke audit data extracts could boost recruitment; however, this strategy was not successful.^30^

As in many other randomised trials of intracerebral haemorrhage, most participants were male, which might be because of their propensity to be invited or consent rather than differences in incidence or outcome of intracerebral haemorrhage compared with women.^31^, ^32^ Although we did not mask the assigned treatment to participants and physicians, the outcomes were objective and adjudicated masked to treatment allocation, which minimises bias.^33^ Antiplatelet regimens used were mostly monotherapy, so the effects of dual antiplatelet therapy remain uncertain. Adherence to the allocated treatment declined over time but was more than 80% even after 4 years of follow-up. Although the sample size was smaller than intended and multiple statistical comparisons were done, we prespecified our primary outcome and main hypothesis, and regarded analyses of secondary outcomes and effects in subgroups as exploratory.

Platelets are the dominant contributor to thrombus formation in the arterial circulation, so antiplatelet therapy predominantly prevents arterial thrombosis. We included venous occlusive events in our composite secondary outcome of all haemorrhagic or occlusive vascular events because randomised trials suggested that antiplatelet therapy might prevent venous occlusive events,^34^ but this benefit was not evident in this trial. However, in this trial, antiplatelet therapy did reduce a composite of major vascular events used by the Antithrombotic Trialists' Collaboration (that did not include venous occlusive events), with a proportionate reduction similar to the effects of aspirin for secondary prevention in their meta-analysis.^4^

Our finding that antiplatelet therapy might have reduced the risk of recurrent intracerebral haemorrhage was unexpected. Although we cannot rule out a random effect, this observation might not be as counterintuitive as it first seems. First, arterial thrombosis can trigger haemorrhage.^35^ Second, more spontaneous intracerebral haemorrhages than expected might be due to haemorrhagic transformation of ischaemic stroke. Finally, inflammation might be a key mechanism underlying intracerebral haemorrhage (as is thought to be the case for intracranial aneurysms). These potential mechanisms underlying RESTART's findings merit further investigation.

Our findings have several implications for future research. We will continue follow-up of the surviving participants in RESTART for another 2 years to improve precision of effect estimates, especially after 2 years of follow-up, and observe whether adherence changes after the trial result is known. Ongoing randomised trials, such as RESTART-Fr (NCT02966119, intended sample size 280) and STATICH (NCT03186729, intended sample size 250), might help to confirm or refute the effects of antiplatelet therapy seen in RESTART.^7^ A prospectively planned individual patient data meta-analysis of RESTART and these trials, and in due course a larger randomised trial, could increase power to detect the overall effects of antiplatelet therapy in these patients and in subgroups of interest with further investigation at earlier times after intracerebral haemorrhage and of heterogeneity of treatment effect by imaging features.^29^ RESTART's findings also support the conduct of randomised trials of oral anticoagulation for survivors of intracerebral haemorrhage with atrial fibrillation, for whom there is some justification for the use of antiplatelet therapy as a comparator.^36^

In summary, RESTART excluded all but a very modest increase in the risk of recurrent intracerebral haemorrhage with antiplatelet therapy, which seemed too small to exceed the established benefits of antiplatelet therapy for secondary prevention of major vascular events (video). Antiplatelet therapy might have reduced the recurrence of intracerebral haemorrhage. These findings provide reassurance about the use of antiplatelet therapy for similar patients in clinical practice. Ongoing randomised trials, their meta-analysis with RESTART, and an adequately powered definitive randomised trial should be done to strengthen the evidence.

## Data sharing

A fully anonymised version of the dataset used for analysis with individual participant data and a data dictionary will be available for other researchers to apply for use 1 year after publication, via https://datashare.is.ed.ac.uk/handle/10283/3265. Written proposals will be assessed by members of the RESTART trial steering committee and a decision made about the appropriateness of the use of data. A data sharing agreement will be put in place before any data are shared.
