# Supplementary material for: Effects of antiplatelet therapy after stroke due to intracerebral haemorrhage (RESTART): a randomised, open-label trial
Source: Lancet. 2019 Jun 29;393(10191):2613–23. doi: 10.1016/S0140-6736(19)30840-2 (PMC6617509; doi:10.1016/S0140-6736(19)30840-2)
Supplement: Supplementary appendix [file mmc1.pdf]

THE LANCET

Supplementary appendix

This appendix formed part of the original submission and has been peer reviewed. We post it as supplied by the authors.

Supplement to: RESTART Collaboration. Effects of antiplatelet therapy after stroke due to intracerebral haemorrhage (RESTART): a randomised, open-label trial. *Lancet* 2019; published online May 22. http://dx.doi.org/10.1016/S0140-6736(19)30840-2.

Effects of antiplatelet therapy after stroke due to intracerebral haemorrhage: a randomised, open-label trial

Online appendix

Appendix of collaborators on delegation logs at hospital sites that recruited at least one participant to RESTART

Sites are listed in descending order of cumulative recruitment (quantified in square brackets), indicating which people took the role of principal investigator (PI).

Edinburgh Royal Infirmary, Edinburgh [39] (Prof. R Al-Shahi Salman (PI), Prof. G Mead, S Burgess, C Lerpiniere, R O'Brien, R Paulton, F Doubal, K McCormick, N Hunter, P Taylor, R Parakramawansha, J Perry, G Blair, A MacRaild); **Salford Royal Foundation Trust, Manchester [21]** (A Parry-Jones (PI), K Shaw, I Burger, A Ingham, T Marsden, J Morell, Z Naing, J Perez, A Hall, R Jarapa, E Wood, V O'Loughlin, S Marshall, L Harrison, M Punter, S Lee, M Johnes); **Northwick Park Hospital, Harrow [18]** (D Cohen (PI), S Davies, K Njoku, M Mpelembue, L Burgess, R Licenik, M Ngwako, N Nisar, R Niranchanan, T Roganova, R Bathula, J Devine, A David, A Oshodi, F Guo, M Abbdul-saheb, A Chandrakumar, A Chamberlain, R Ballantine, E Owoyele, V Sukdeo, P Poku); **Royal Hallamshire Hospital, Sheffield [13]** (K Harkness (PI), C Blank (PI), P Bayliss, E Richards, K Birchall, O Balitska, A Ali, F Kibutu, C Doyle, J Howe, C Kamara, K Stocks, Prof. A Majid, A Maatouk, L Barron, R Lindert, J Redgrave, K Dakin); **Torbay District General Hospital, Torquay [13]** (B Bhaskaran (PI), S Szabo, I Salih, D Kelly, D Tomlin, H Bearne, P Fitzell, J Buxton, G Ayres, H Bhakri, J Garfield-Smith, K Horan, A Saulat); **Southend University Hospital NHS Foundation Trust, Westcliff on Sea [12]** (P Guyler (PI), D Sinha (PI) T Loganathan, A Siddiqui, L Coward, S Tysoe, S Kunhunny, S Shah, K Ng, N Menon, R Orath Prabakaran, S Kelavkar, S Rashmi, D Ngo); **Monklands Hospital, Airdrie [11]** (M Barber (PI), D Esson, F Brodie); **Morriston Hospital, Swansea [11]** (T Anjum (PI), M Wani (PI), M Krishnan, L Quinn, J Spencer, S Chenna, S Storton, T Jones, H Thompson-Jones, L Dacey, S Thomas, T Beaty, S Treadwell, C Davies, L Connor, S Tucker, G Gainard, P Slade); **University Hospitals of North Midlands NHS Trust, Stoke-on-Trent [11]** (G Muddegowda (PI), R Sanyal (PI), S Stevens, A Butler, R Varquez, A Remegoso, N Abano, F Alipio, H Denic, R Carpio, C Causley, A Moores, S Lyjko, Prof. C Roffe, J Hiden, P Ferdinand, A Barry, H Maguire, J Grocott, K Finney); **Victoria Hospital, Kirkcaldy [11]** (V Cvoro (PI), M Couser, K Ullah, N Chapman, K McCormick, S Mcauley, S Pound); **City Hospital, Nottingham [10]** (S Raghunathan (PI), F Shelton, A Hedstrom, N Gilzeane, J Roffe, J Clarke, D Havard, A Buck, K Krishnan, M Godfrey, N Sprigg, S Sheikh, K Whittamore, R Keshvara, B Jackson, J Appleton, Z Law, O Matias, G Wilkes, C Jordan); **Hillingdon Hospital, Uxbridge [10]** (E Vasileiadis (PI), C Mason, A Parry, G Landers, M Holden, B Aweid); **Yeovil District Hospital, Yeovil [10]** (K Rashed (PI), L Balian, C Vickers, B Williams-Yesson, E Keeling, S Board, J Allison, C Buckley, J Board, D Wood, T Pitt-Kerby, A Tanate); **Doncaster Royal Infirmary, Doncaster [9]** (M Kini (PI), D Walstow, D Chadha, R Fong); **North Middlesex University Hospital, London [9]** (R Luder (PI), T Adesina, J Gallagher, M Bhargava, C van Someren, E Murali, H Bridger); **Royal Cornwall Hospital,**

Truro [9] (F Harrington (PI), A James, K Adie, A Mate, G Courtauld, C Schofield, K Bond, L Lucas, B Maund, S Ellis); **Royal Devon & Exeter Hospital, Exeter [9]** (P Mudd (PI), M James, S Keenan, A Bowring, J Cageao, D Strain, H Kingwell, C Roughan, A Hemsley, J Sword, K Miller, A Goff, K Gupwell, K Thorpe); **Royal Preston Hospital, Preston [9]** (H Emsley (PI), S Punekar (PI), A McLoughlin, S Sultan, B Gregory, S Raj, D Doyle); **Queen Elizabeth University Hospital, Glasgow [9]** (Prof. K Muir (PI), W Smith, N Day, A Welch, F Moreton, B Cheripelli, D Kalladka, X Huang, S El Tawil, S Ramachandran, C Crosbie, J Elliot); **Guys & St Thomas, London [8]** (Prof. T Rudd (PI), A Bhalla, J Birns, K Marks, S Kullane); **Southampton General Hospital, Southampton [8]** (N Weir (PI), C Allen, V Pressly, E Battersby-Wood, P Crawford, S Egerton, A Blades, G Howard, J Marigold, S Evans, A Walters, F Smith, I Gartrell, C Cox, R Creeden, S Smith, C Boxall); **Ystrad Mynach Hospital, Ystrad Mynach, Newport [8]** (J Hewitt (PI), C Nott, S Procter, S Buckle, J Whiteman, C Triscott, R Mardania, R Wallace, J Gray); **Calderdale Royal Hospital, Halifax [7]** (A Nair (PI), J Greig, P Rana, M Robinson, M Alam); **University College London Hospital, London [7]** (Prof D Werring (PI), I Jones, A Banaras, L Crook, C Watchurst, M Brezitski, K Patel, D Wilson, R Erande, C Hogan, N Oji, N Francia, A Ashton, S Feerick, I Hostettler, T Al-Mayhani, E Elliott); **Altnagelvin Hospital, Derry/Londonderry [6]** (M McCarron (PI), J McKee, M Doherty, F McVerry, C Blair); **Bristol Royal Infirmary, Bristol [6]** (C Holmes (PI), S Caine (PI), M Osborn, E Dodd, P Murphy, N Devitt, P Baker, A Steele, L Guthrie, S Clarke); **Gloucestershire Royal Hospital, Gloucester [6]** (D Dutta (PI), P Brown, D Ward, F Davis, J Turfrey, R Bakawala, C Hughes, K Collins, S O'Connell, J Glass); **James Cook University Hospital, Middlesbrough [6]** (D Broughton (PI), D Tryambake (PI), L Dixon, K Chapman, A Young, A Bergin, A Sigsworth); **Kings Mill Hospital, Mansfield [6]** (M Cooper (PI), M Nasar, I Wynter, A Rajapakse); **Leeds General Infirmary, Leeds [6]** (A Hassan (PI), M Kambafwile, L Makawa, D Waugh, E Veraque, M Randall, V Papavasileiou); **Royal Liverpool and Broadgreen University Hospital, Liverpool [6]** (A Manoj (PI), M Wilkinson, G Fletcher, P Lopez, P Cox, P Fitzsimmons, N Sharma); **Royal United Hospital, Bath [6]** (J Choulerton (PI), B Madigan, D Button, L Dow, L Gbadamoshi, J Avis, S McCann, L Shaw, D Howcroft, S Lucas, A Stone); **St Georges Healthcare NHS Trust, London [6]** (G Cluckie (PI), C Lovelock (PI), B Patel, B Clarke, N Chopra, K Kennedy, R Williams, L Kerin, N Jeyaraj, L Choy, N Clarke, F Watson, S Trippier, B Moynihan, U Khan, N Dayal, C Orefo, T Adedoyin, R Ghatala, A Blight, V Jones, J O'Reilly); **The Royal Bournemouth Hospital, Bournemouth [6]** (K Thavanesan (PI), D Tiwari (PI), C Cox, J Roberts, B Jupp, M Keltos, A Iqbal, C Bagnall, L Tucker, A Ljubez, O David, E Rogers, C Ovington, J Bell, B Longland, G Hann); **University Hospital Aintree, Liverpool [6]** (C Cullen (PI), H Thant, T Ingram, M Zoe, J Peters, V Sutton, R Durairaj, D Shackcloth, J Ewing, S Stevenson, M Harrison); **University Hospital of North Tees, Stockton [6]** (I Anwar (PI), B Kumar, H Skinner, T Nozedar, D McArdle, S Crawford, A Annamalai, A Ramshaw); **Western General Hospital, Edinburgh [6]** (Prof. M Dennis (PI), Prof. C Sudlow, W

Whiteley, C Lerpiniere, Prof. R Al-Shahi Salman, R Fraser); **Aberdeen Royal Infirmary, Aberdeen [5]** (M MacLeod (PI), J Irvine, A Joyson, H Gow, J Furnace, B Jagpal, S Ross, S Nelson, R Clarke, N Crouch, K Klaasen, B MacLennan, V Taylor); **Addenbrooke's Hospital, Cambridge [5]** (E O'Brien (PI), S Finlay, H Hayhoe, D Handley, S Kelly, J Francis, N Hannon, G Zachariah, J Mcgee, J Mitchell, E Amis, J Sesay, S Crisp); **Barnet Hospital, Barnet [5]** (D Epstein (PI), A Shukla, I Jones, V Krishnamurthy, P Nicholas, A Webber, S Qureshi, J Penge); **Bradford Royal Infirmary, Bradford [5]** (H Ramadan (PI), S Maguire, C Patterson, R Bellfield, B Hairsine, O Quinn, M Hooley, K Stewart); **John Radcliffe Hospital, Oxford [5]** (U Schulz (PI), R Teal, P Mathieson, I Reckless, J Kennedy, Prof. G Ford, G Lenti, G Harston); **Nevill Hall Hospital, Abergavenny [5]** (B Richard (PI), S Buckle, S Procter, S Moseley, C Nott, J Whiteman, C Triscott, R Wallace, M Edwards, H Lawson, M Tayler); **The Royal London Hospital, London [5]** (T Harrison (PI), K Saastamoinen (PI), A Salek-Haddadi, D Hove, L Howaniec, G Grimwood, O Redjep, F Humphries, S Amlani, L Cuenoud, E Erumere, G Auld, L Argandona); **University Hospital North Durham, Durham [5]** (Y Pai (PI), M Dhakal (PI), S Dima (PI), B Esisi (PI), G Smith, M Garside, D Bruce, R Hayman, S Clayton, E Brown, G Rogers, M Naeem, V Baliga); **University Hospital of Wales, Cardiff [5]** (T Hughes (PI), B Morse, S White, S Schwarz, E Tallantyre, A Osman, H De Berker, B Jelley); **Wythenshawe Hospital, Manchester [5]** (E Gamble (PI), B Charles, R Grue, A Chaudhry, S Blane, A Hague, C Lambert); **Ayr Hospital, Ayr [4]** (S Ghosh (PI), D Gilmour, E Barrie, M Henry); **Charing Cross Hospital, London [4]** (M Venter (PI), A Kar (PI), S Mashate, K Harvey, L Gardener, V Nguyen, B Hazel, O Geraghty, O Halse, P Wilding, V Tilley); **Derby Royal Hospital, Derby [4]** (Prof. T England (PI), A Hedstrom, M Maddula, Prof. R Donnelly); **Heartlands Hospital, Birmingham [4]** (R Yadava (PI), K Azhar (PI), M Sangombe, J Reddan, S Stafford); **New Cross Hospital, Wolverhampton [4]** (K Fotherby (PI), D Morgan, F Baig, K Jennings-Preece, D Butler, N Ahmad, B Rai, A Stevens, A Willberry); **Queen Alexandra Hospital, Portsmouth [4]** (P Siddegowda (PI), L Hyatt, A Saulat, J Tandy, P Howard, T Dobson, D Jarrett, S Ponnambath, S Valentine, C James, R Butler, Y Harrington-Davies, A Suttling); **Queen Elizabeth Hospital, Gateshead [4]** (B Esisi (PI), T Cassidy (PI), B McClelland, M Bokhari); **Raigmore Hospital, Inverness [4]** (P Findlay (PI), A Macaden, I Shread, C Barr); **Royal Infirmary, Glasgow [4]** (Prof. P Langhorne (PI), G Kerr, F Wright, R Graham, C McAlpine, L Humphreys, M Iqbal); **Royal Surrey County Hospital, Guildford [4]** (K Pasco (PI), O Balazikova, A Nasim, C Peixoto, S Shahmehri, L Gallagher); **William Harvey Hospital, Ashford [4]** (T Webb (PI), L Cowie, A Thomson, H Rudenko, A Verrion, E Beranova, T Cosier, S Walker, S McDonald, N Schumacher); **Derriford Hospital, Plymouth [3]** (A Mohd Nor (PI), C Eglinton, N Persad, C Brown, M Weinling, A Shah, J Baker, B Hyams); **Forth Valley Royal Hospital, Larbert [3]** (A Byrne (PI), C McGhee, A Smart, C Copeland); **Hull Royal Infirmary, Hull [3]** (R Rayessa (PI), L Wilson, C Naylor, A Rodgers, S Wilson, E Clarkson); **Pinderfields Hospital, Wakefield [3]** (M Carpenter (PI), M Walker, R Davey, A

Needle, R Fathima, G Bateman, A Stanners, P Datta, L Jackson, J Ball); **Royal Victoria Infirmary, Newcastle upon Tyne [3]** (M Davis (PI), H Guy, N Atkinson, M Fawcett, T Thompson, C Hays, S Woodward, V Hogg); **Salisbury District Hospital, Salisbury [3]** (T Black (PI), A Anthony, S Miriam, C Clarke, D Mead, M Tribbeck, J Cronin, R Fennelly); **St Mary's Hospital, Newport Isle of Wight [3]** (M Haque (PI), E Hakim (PI), S Symonds, M Maanoosi, J Herman); **St Richards Hospital, Chichester [3]** (S Ivatts (PI), Y Baird, M Sally, I Amey, L Clayton- Evans, S Newton, I Chadbourn); **Victoria Hospital, Blackpool [3]** (J McIlmoyle (PI), C Jeffs, C Dickinson, J Howard, S Anwar, S Dhar, K Jones, M Siddiq, C Clay); **Arrowe Park Hospital, Wirral [2]** (R Davies (PI), P Owings, G Sangster, V Gott, V Little, P Weir, S Cherian, D Jose, H Moroney, S Downham, A Dodd, L Codd, V Vettimootal Johnson, N Robinson); **Barnsley Hospital NHS Foundation Trust, Barnsley [2]** (A Ahmed (PI), M Albazzaz (PI), S Johnson, C Denniss, T Zahoor, M Cunningham); **Countess of Chester NHS Foundation Trust, Chester [2]** (T Webster (PI), K Chatterjee, A Nallasivan, S Haider, S Leason, C Perkins, S Seagrave); **Hereford County Hospital, Hereford [2]** (C Jenkins (PI), F Price, C Hughes, L Mercer); **Leicester Royal Infirmary, Leicester [2]** (D Eveson (PI), A Mistri, L Manning, C Patel, M Moqsith, S Khan, C Stephens, S Sattar, M Lam, K Musarrat); **Leighton Hospital, Crewe [2]** (L Kalathil (PI), R Miller, M Salehin, N Gautam, D Bailey, K Amor, J Meir); **Luton & Dunstable NHSFT University Hospital, Luton [2]** (L Sekaran (PI), F Justin, M Tate, K Bharaj, R Simon, N Mohammed, S Sethuraman, D Phiri, M Chauhan); **Musgrove Park Hospital, Taunton [2]** (M Hussain (PI), S Brown, M Harvey, R Whiting, M Khan, J Homan, L Foote, N Hunt, A Whitcher, C Pawley, E Foster, J Foot, H Durman, L Brotherton); **Norfolk & Norwich University Hospital, Norwich [2]** (K Metcalf (PI), J Jagger, S McDonald, K Waterfield, P Sutton, J Saada, A Wiltshire, R Perfitt, R Greenwood, N Shinh, A Anversha, G Ravenhill); **Pilgrim Hospital, Boston [2]** (D Mangion (PI), S Markova, A Hardwick, T Lawrence, J Fletcher, C Constantin, K Pettitt, I Thomas); **Queens Hospital, Romford [2]** (S Andole (PI), N Gadapa, K Dunne, M Krommyda, E Burssens, C Plewa, S King); **Royal Hampshire County Hospital, Winchester [2]** (N Smyth (PI), J Wilson, E Giallombardo, C Eglinton, L Sykes); **Royal Lancaster Infirmary, Lancaster [2]** (P Kumar (PI), P Thomas, l Dunn, C Culmsee, I Huggett, J Barker); **Royal Victoria Hospital, Belfast [2]** (I Wiggam (PI), A Wallace, E Kerr, A Fulton, A Hunter, S Tauro, S Cuddy); **Solihull Hospital, Solihull [2]** (K Elfandi (PI), U Khan, S Stafford, J Reddan); **Sunderland Royal Hospital, Sunderland [2]** (M Myint (PI), R O'Brien (PI), H Brew, N Majmudar, J OConnell, G Bunea, C Fox, D Gulliver, N Sattar, B Mokoena, A Smith, E Osborne, R Krishnamurthy); **Ulster Hospital, Belfast [2]** (D Wilson (PI), B Wroath, K Dynan, M Power, S Thompson, V Adell); **West Cumberland Hospital, Whitehaven [2]** (E Orugun (PI), U Poultney, H Crowther, R Glover, S Thornthwaite); **West Suffolk Hospital, Bury St Edmunds [2]** (A Nicolson (PI), L Wood, J Imam, J White); **Bedford Hospital, Bedford [1]** (H Ni (PI), C Graham, B Rahman, J Milligan, J Jose); **Chesterfield Royal Hospital, Chesterfield [1]** (M Sajid (PI), G Ghaly, M Ball, R Gascoyne); **Dorset County**

Hospital NHS Foundation Trust, Dorchester [1] (H Proeschel (PI), S Sharpe, S Horton, S Jones, E Beaves); **Epsom General Hospital, Epsom [1]** (J Putterill (PI), R Jha, R Gallifent, P Kakar); **Hairmyres Hospital, East Kilbride [1]** (B Yip (PI), M Bell, B MacInnes, L MacLiver, D Esson); **Lister Hospital, Stevenage [1]** (A Pusalkar (PI), K Chan, P Dangri, K Crabtree, H Beadle, A Cook); **Peterborough City Hospital, Peterborough [1]** (S Subramonian (PI), P Owusu-Agyei (PI), N Temple, N Butterworth-Cowin); **Poole Hospital, Poole [1]** (S Ragab (PI), K Knops, E Jinks, C Dickson, L Gleave, J Leggett, J Dube, T Garcia); **Prince Charles Hospital, Merthyr Tydfil [1]** (R Dewar (PI), K Thomas, J White); **Queen Elizabeth Hospital, Birmingham [1]** (D Sims (PI), J Hurley, M Willmot, C Sutton, E Littleton, S Maiden, J Cunningham, R Jones, C Green, M Bates); **Queen Elizabeth Hospital, Kings Lynn [1]** (R Shekhar (PI), R Crown, E Gilham, T Fuller, I Ahmed, K Waterfield); **Royal Blackburn Hospital, Blackburn [1]** (N Goorah (PI), A Bell, C Kelly, A Singh, J Walford, S Duberley, B Tomlinson, F Patel); **Royal Sussex County Hospital, Brighton [1]** (I Kane (PI), N Gainsborough, J Gaylard, J Breeds, Prof. C Rajkumar, S Hervey, A Pitt-Ford, L Latter, E Barbon, P Thompson); **Sandwell General Hospital, Birmingham [1]** (S Ispoglou (PI), R Evans, S Ankolekar, A Hayes); **South West Acute Hospital, Enniskillen [1]** (B Keegan (PI), M Doherty, J Kelly, C Blair); **Stepping Hill Hospital, Stockport [1]** (S Krishnamoorthy (PI), J Vassallo, D Walter, H Cochrane); **The Princess Royal Hospital, Telford [1]** (M Srinivasan (PI), F Hurford, D Donaldson, R Campbell, N Motherwell, I Mukherjee); **University Hospitals Coventry and Warwickshire, Coventry [1]** (A Kenton (PI), S Nyabadza, I Martin, B Hunt, H Hassan, B Dallol, S O'Toole).

Literature search strategies used to put the research in context

CENTRAL search strategy

#1 MESH DESCRIPTOR Basal Ganglia Hemorrhage EXPLODE ALL TREES

#2 MESH DESCRIPTOR Intracranial Hemorrhages

#3 MESH DESCRIPTOR Intracranial Hemorrhage, Hypertensive

#4 MESH DESCRIPTOR Cerebral Hemorrhage

#5 (brain* or cerebr* or cerebell* or intracerebral or intracran* or parenchymal or intraparenchymal or intraventricular or infratentorial or supratentorial or putaminal or putamen or hemispher* or stroke or apoplex*):TI

#6 (basal and gangli*):TI

#7 (posterior and fossa):TI,AB,KY

#8 (haemorrhag* or hemorrhag* or haematoma* or hematoma* or bleed*):TI

#9 #5 or #6 or #7 and #8

#10 (ICH or ICHs):TI

#11 #1 or #2 or #3 or #4 or #9 or #10

#12 MESH DESCRIPTOR Anticoagulants EXPLODE ALL TREES

#13 MESH DESCRIPTOR Pipecolic Acids EXPLODE ALL TREES WITH QUALIFIERS AE,TU

#14 MESH DESCRIPTOR Vitamin K EXPLODE ALL TREES

#15 MESH DESCRIPTOR Thrombin EXPLODE ALL TREES WITH QUALIFIERS AI

#16 MESH DESCRIPTOR Factor Xa

#17 MESH DESCRIPTOR Blood Coagulation Factors EXPLODE ALL TREES WITH QUALIFIERS AI

#18 MESH DESCRIPTOR Blood Coagulation EXPLODE ALL TREES WITH QUALIFIERS DE

#19 MESH DESCRIPTOR Antithrombins EXPLODE ALL TREES

#20 MESH DESCRIPTOR Hirudin Therapy EXPLODE ALL TREES

#21 (anticoagul* or antithromb*):TI,AB,KY

#22 (Vitamin next K next antagonist*):TI,AB,KY

#23 (VKA or VKAs):TI,AB,KY

#24 #22 or #23

#25 (direct* NEAR5 thrombin):TI,AB,KY

#26 "DTI":TI,AB,KY

#27 (factor next Xa NEAR5 inhib*):TI,AB,KY

#28 (factor next 10a NEAR5 inhib*):TI,AB,KY

#29 (fXa NEAR5 inhib*):TI,AB,KY

#30 (autoprothrombin NEAR5 inhib*):TI,AB,KY

#31 (thrombokinase NEAR5 inhib*):TI,AB,KY

#32 (acenocoumarol* or dicoumarol* or ethyl next biscoumacetate* or phenprocoumon* or warfarin* or ancrod* or citric next acid* or coumarin* or chromonar* or coumestro* or esculi* or ochratoxin* or umbelliferone* or dermatan next sulfate* or dextran* or edetic next acid* or enoxaparin* or gabexate* or heparin* or lmwh* or nadroparin* or pentosan next sulfuric next polyester* or phenindione* or protein next c or protein next s or tedelparin*):TI,AB,KY

#33 (tinzaparin or parnaparin or dalteparin or reviparin or danaparoid or lomoparan or org next 10172 or mesoglycan or polysaccharide next sulphate* or sp54 or sp-54 or md805 or md-805 or cy222 or cy-222 or cy216 or cy-216):TI,AB,KY

#34 (Marevan or Fragmin* or Fraxiparin* or Klexane):TI,AB,KY

#35 (argatroban or MD805 or MD-805 or dabigatran or ximelagatran or melagatran or efegatran or flovagatran or inogatran or napsagatran or bivalirudin or lepirudin or hirudin* or desirudin or desulfatohirudin or hirugen or hirulog or AZD0837 or bothrojaracin or odiparcil):TI,AB,KY

#36 (xabans or antistasin or apixaban or betrixaban or du next 176b or eribaxaban or fondaparinux or idraparinux or otamixaban or razaxaban or rivaroxaban or yagin or ym next 150 or ym150 or LY517717):TI,AB,KY

#37 #25 or #26 or #27 or #28 or #29 or #30 or #31 or #32 or #33 or #34 or #35 or #36

#38 MESH DESCRIPTOR Platelet Glycoprotein GPIIb-IIIa Complex EXPLODE ALL TREES WITH QUALIFIERS AI,DE

#39 MESH DESCRIPTOR Platelet Activation EXPLODE ALL TREES WITH QUALIFIERS DE

#40 MESH DESCRIPTOR Blood Platelets EXPLODE ALL TREES WITH QUALIFIERS DE

#41 (antiplatelet* or anti-platelet* or antiaggreg* or anti-aggreg* or (platelet* NEAR5 inhibit*) or (thrombocyt* NEAR5 inhibit*)):TI,AB,KY #42 (alprostadil* or aspirin* or acetylsalicylic next acid or (acetyl ADJ salicylic and acid*) or (acetyl-salicylic and acid or epoprostenol* or ketanserin* or ketorolac next tromethamine* or milrinone* or mopidamol* or procainamide* or thiophen* or trapidil* or picotamide* or ligustrazine* or levamisol* or suloctidil* or ozagrel* or oky046 or oky-046 or defibrotide* or cilostazol or satigrel or sarpolgrelate or kbt3022 or kbt-3022 or isbogrel or cv4151 or cv-4151)):TI,AB,KY

#43 ((glycoprotein next iib* near/5 inhib*) or (glycoprotein next iib* near/5 antag*) or (gp next iib* near/5 inhib*) or (gp next iib* near/5 antag*) or GR144053 or GR-144053 or triflusal):TI,AB,KY

#44 (Argatroban or Beraprost or Cicaprost or Cilostazol or Clopidogrel or Dipyridamole or Iloprost or Indobufen or Lepirudin or Pentosan next Polysulfate or Pentoxifylline or Piracetam or Prostacyclin or Sulfinpyrazone or Sulphinpyrazone or Ticlopidine or Triflusal or Abciximab or Disintegrin or Echistatin or Eptifibatide or Lamifiban or Orbofiban or Roxifiban or Sibrafiban or Tirofiban or Xemilofiban or terutroban or picotamide or prasugrel):TI,AB,KY

#45 (Dispril or Albyl* or Ticlid* or Persantin* or Plavix or ReoPro or Integrilin* or Aggrastat):TI,AB,KY

#46 MESH DESCRIPTOR Platelet Aggregation Inhibitors EXPLODE ALL TREES

#47 #38 or #39 or #40 or #41 or #42 or #43 or #44 or #45 or #46

#48 #37 or #47

#49 #48 and #11

MEDLINE search strategy

1. exp basal ganglia haemorrhage/ or intracranial hemorrhages/ or cerebral haemorrhage/ or intracranial haemorrhage, hypertensive/

2. ((brain$ or cerebr$ or cerebell$ or intracerebral or intracran$ or parenchymal or intraparenchymal or intraventricular or infratentorial or supratentorial or basal gangli$ or putaminal or putamen or posterior fossa or hemispher$) adj5 (h?emorrhag$ or h?ematoma$ or bleed$)).tw.

3. ((h?emorrhag$ or bleed$) adj5 (stroke or apoplex$)).tw.

4. (ICH or ICHs).tw.

5. 1 or 2 or 3 or 4

6. exp anticoagulants/

7. exp Vitamin K/ai or thrombin/ai or factor Xa/ai or exp Blood coagulation factors/ai

8. exp antithrombins/ or hirudin therapy/

9. (anticoagul$ or antithromb$).tw.

10. (Vitamin K antagonist$ or VKA or VKAs).tw.

11. (direct$ adj3 thrombin adj3 inhib$).tw.

12. DTI$1.tw.

13. ((factor Xa or factor 10a or fXa or autoprothrombin c or thrombokinase) adj3 inhib$).tw.

14. (activated adj3 (factor X or factor 10) adj3 inhib$).tw.

15. (acenocoumarol$ or dicoumarol$ or ethyl biscoumacetate$ or phenprocoumon$ or warfarin$ or ancrod$ or citric acid$ or coumarin$ or chromonar$ or coumestro$ or esculi$ or ochratoxin$ or umbelliferone$ or dermatan sulfate$ or

dextran$ or edetic acid$ or enoxaparin$ or gabexate$ or heparin$ or lmwh$ or nadroparin$ or pentosan sulfuric polyester$ or phenindione$ or protein c or protein s or tedelparin$).tw,nm.

16. (tinzaparin or parnaparin or dalteparin or reviparin or danaparoid or lomoparan or org 10172 or mesoglycan or polysaccharide sulphate$ or sp54 or sp-54 or md805 or md-805 or cy222 or cy-222 or cy216 or cy-216).tw,nm.

17. (Marevan or Fragmin$ or Fraxiparin$ or Klexane).tw,nm.

18. (argatroban or MD805 or MD-805 or dabigatran or ximelagatran or melagatran or efegatran or flovagatran or inogatran or napsagatran or bivalirudin or lepirudin or hirudin$ or desirudin or desulfatohirudin or hirugen or hirulog or AZD0837 or bothrojaracin or odiparcil).tw,nm.

19. (xabans or antistasin or apixaban or betrixaban or du 176b or eribaxaban or fondaparinux or idraparinux or otamixaban or razaxaban or rivaroxaban or yagin or ym 150 or ym150 or LY517717).tw,nm.

20. exp platelet aggregation inhibitors/ or exp platelet glycoprotein gpiib-iiia complex/ai

21. (antiplatelet$ or anti-platelet$ or antiaggreg$ or anti-aggreg$ or (platelet$ adj3 inhibit$) or (thrombocyt$ adj3 inhibit$)).tw.

22. (alprostadil$ or aspirin$ or acetylsalicylic acid or acetyl salicylic acid$ or acetyl?salicylic acid or epoprostenol$ or ketanserin$ or ketorolac tromethamine$ or milrinone$ or mopidamol$ or procainamide$ or thiophen$ or trapidil$ or picotamide$ or ligustrazine$ or levamisol$ or suloctidil$ or ozagrel$ or oky046 or oky-046 or defibrotide$ or cilostazol or satigrel or sarpolgrelate or kbt3022 or kbt-3022 or isbogrel or cv4151 or cv-4151 or ((glycoprotein iib$ or gp iib$) adj5 (antagonist$ or inhibitor$)) or GR144053 or GR-144053 or triflusal).tw,nm.

23. (Beraprost or Cicaprost or Cilostazol or Clopidogrel or Dipyridamole or Iloprost or Indobufen or Lepirudin or Pentosan Polysulfate or Pentoxifylline or Piracetam or Prostacyclin or Sulfinpyrazone or Sulphinpyrazone or Ticlopidine or Triflusal or Abciximab or Disintegrin or Echistatin or Eptifibatide or Lamifiban or Orbofiban or Roxifiban or Sibrafiban or Tirofiban or Xemilofiban or terutroban or picotamide or prasugrel).tw,nm.

24. (Dispril or Albyl$ or Ticlid$ or Persantin$ or Plavix or ReoPro or Integrilin$ or Aggrastat).tw,nm.

25. or/6-24

26. Randomized Controlled Trials as Topic/

27. random allocation/

28. Controlled Clinical Trials as Topic/

29. control groups/

30. clinical trials as topic/ or clinical trials, phase i as topic/ or clinical trials, phase ii as topic/ or clinical trials, phase iii as topic/ or clinical trials, phase iv as topic/

31. double-blind method/

32. single-blind method/

33. Placebos/

34. placebo effect/

35. randomised controlled trial.pt.

36. controlled clinical trial.pt.

37. (clinical trial or clinical trial phase i or clinical trial phase ii or clinical trial phase iii or clinical trial phase iv).pt.

38. (random$ or RCT or RCTs).tw.

39. (controlled adj5 (trial$ or stud$)).tw.

40. (clinical$ adj5 trial$).tw.

41. ((control or treatment or experiment$ or intervention) adj5 (group$ or subject$ or patient$)).tw.

42. (quasi-random$ or quasi random$ or pseudo-random$ or pseudo random$).tw.

43. ((control or experiment$ or conservative) adj5 (treatment or therapy or procedure or manage$)).tw.

44. ((singl$ or doubl$ or tripl$ or trebl$) adj5 (blind$ or mask$)).tw.

45. (placebo$ or sham).tw.

46. trial.ti.

47. (assign$ or allocat$).tw.

48. or/26-47

49. 5 and 25 and 48

50. exp animals/ not humans/

51. 49 not 50

EMBASE (Ovid) search strategy

1. anticoagulant agent/ or antivitamin k/ or exp blood clotting inhibitor/ or exp coumarin anticoagulant/ or defibrotide/ or dextran sulfate/ or fluindione/ or glycosaminoglycan polysulfate/ or exp heparin derivative/ or lupus anticoagulant/ or phenindione/

2. (anticoagul$ or antithromb$).tw.

3. (Vitamin K antagonist$ or VKA or VKAs).tw.

4. (direct$ adj5 thrombin adj5 inhib$).tw.

5. DTI$1.tw.

6. ((factor Xa or factor 10a or fXa or autoprothrombin c or thrombokinase) adj5 inhib$).tw.

7. (activated adj5 (factor X or factor 10) adj5 inhib$).tw.

8. (acenocoumarol$ or dicoumarol$ or ethyl biscoumacetate$ or phenprocoumon$ or warfarin$ or ancrod$ or citric acid$ or coumarin$ or chromonar$ or coumestro$ or esculi$ or ochratoxin$ or umbelliferone$ or dermatan sulfate$ or dextran$ or edetic acid$ or enoxaparin$ or gabexate$ or heparin$ or lmwh$ or nadroparin$ or pentosane sulfuric polyester$ or phenindione$ or protein c or protein s or tedelparin$).tw.

9. (tinzaparin or parnaparin or dalteparin or reviparin or danaparoid or lomoparan or org 10172 or mesoglycan or polysaccharide sulphate$ or sp54 or sp-54 or md805 or md-805 or cy222 or cy-222 or cy216 or cy-216).tw.

10. (Marevan or Fragmin$ or Fraxiparin$ or Klexane).tw.

11. (argatroban or MD805 or MD-805 or dabigatran or ximelagatran or melagatran or efegatran or flovagatran or inogatran or napsagatran or bivalirudin or lepirudin or hirudin$ or desirudin or desulfatohirudin or hirugen or hirulog or AZD0837 or bothrojaracin or odiparcil).tw.

12. (xabans or antistasin or apixaban or betrixaban or du 176b or eribaxaban or fondaparinux or idraparinux or otamixaban or razaxaban or rivaroxaban or yagin or ym 150 or ym150 or LY517717).tw.

13. or/1-12

14. exp antithrombocytic agent/

15. fibrinogen receptor/dt [Drug Therapy]

16. (antiplatelet$ or anti-platelet$ or antiaggreg$ or anti-aggreg$ or (platelet$ adj5 inhibit$) or (thrombocyt$ adj5 inhibit$)).tw.

17. (alprostadil$ or aspirin$ or acetylsalicylic acid or acetyl salicylic acid$ or acetyl?salicylic acid or epoprostenol$ or ketanserin$ or ketorolac tromethamine$ or milrinone$ or mopidamol$ or procainamide$ or thiophen$ or trapidil$ or picotamide$ or ligustrazine$ or levamisol$ or suloctidil$ or ozagrel$ or oky046 or oky-046 or defibrotide$ or cilostazol or satigrel or sarpolgrelate or kbt3022 or kbt-3022 or isbogrel or cv4151 or cv-4151 or ((glycoprotein iib$ or gp iib$) adj5 (antagonist$ or inhibitor$)) or GR144053 or GR-144053 or triflusal).tw.

18. (Argatroban or Beraprost or Cicaprost or Cilostazol or Clopidogrel or Dipyridamole or Iloprost or Indobufen or Lepirudin or Pentosan Polysulfate or Pentoxifylline or Piracetam or Prostacyclin or Sulfinpyrazone or Sulphinpyrazone or Ticlopidine or Triflusal or Abciximab or Disintegrin or Echistatin or Eptifibatide or Lamifiban or Orbofiban or Roxifiban or Sibrafiban or Tirofiban or Xemilofiban or terutroban or picotamide or prasugrel).tw.

19. (Dispril or Albyl$ or Ticlid$ or Persantin$ or Plavix or ReoPro or Integrilin$ or Aggrastat).tw.

20. or/14-19

21. 13 or 20

22. *basal ganglion hemorrhage/ or *brain hemorrhage/ or *brain ventricle hemorrhage/ or *cerebellum hemorrhage/

23. ((brain$ or cerebr$ or cerebell$ or intracerebral or intracran$ or parenchymal or intraparenchymal or intraventricular or infratentorial or supratentorial or basal gangli$ or putaminal or putamen or posterior fossa or hemispher$ or stroke or apoplex$) adj5 (h?emorrhag$ or h?ematoma$ or bleed$)).ti.

24. 22 or 23 or (ICH or ICHs).ti.

25. randomized controlled trial/ or "randomized controlled trial (topic)"/

26. Randomization/

27. Controlled Study/

28. control group/

29. clinical trial/ or phase 1 clinical trial/ or phase 2 clinical trial/ or phase 3 clinical trial/ or phase 4 clinical trial/ or controlled clinical trial/

30. Double Blind Procedure/

31. Single Blind Procedure/ or triple blind procedure/

32. placebo/

33. drug comparison/ or drug dose comparison/

34. "types of study"/

35. random$.tw.

36. (controlled adj5 (trial$ or stud$)).tw.

37. (clinical$ adj5 trial$).tw.

38. ((control or treatment or experiment$ or intervention or surgical) adj5 (group$ or subject$ or patient$)).tw.

39. (quasi-random$ or quasi random$ or pseudo-random$ or pseudo random$).tw.

40. ((singl$ or doubl$ or tripl$ or trebl$) adj5 (blind$ or mask$)).tw.

41. placebo$.tw.

42. controls.tw.

43. or/25-42

44. meta analysis/ or "meta analysis (topic)"/ or "systematic review"/ or "systematic review (topic)"/

45. meta analy$.tw.

46. metaanaly$.tw.

47. (systematic adj (review$1 or overview$1)).tw.

48. literature/

49. (cochrane or embase or psychlit or psyclit or psychinfo or psycinfo or cinahl or cinhal or science citation index or bids or medline or pubmed).ab.

50. (reference list$ or bibliograph$ or hand-search$ or relevant journals or manual search).ab.

51. (selection criteria or data extraction).ab.

52. review.pt. or literature/ or review/

53. 51 and 52

54. 44 or 45 or 46 or 47 or 48 or 49 or 50 or 53

55. (letter or editorial).pt.

56. 54 not 55

57. 43 or 56

58. 21 and 24 and 57

59. limit 58 to human

Trials register search strategies

1. US National Institutes of Health Ongoing Trials Register (ClinicalTrials.gov)

'Randomised' AND 'intracerebral haemorrhage'

2. World Health Organization (WHO) International Clinical Trials Registry Platform (ICTRP) (apps.who.int/trialsearch)

'Intracerebral haemorrhage' OR 'Intracerebral hemorrhage' OR 'ICH'

3. ISRCTN Registry (www.isrctn.com)

'Intracerebral haemorrhage' OR 'Intracerebral hemorrhage' OR 'ICH'

4. Stroke Trials Registry – the Internet Stroke Center (www.strokecenter.org/trials/)

'Intracerebral haemorrhage' OR 'Intracerebral hemorrhage' OR 'ICH'

External assessment of a random sample of internally assessed outcome events

RESTART's primary sources of outcome event ascertainment were: (1) participant/carer and general practitioner postal/telephone follow-up questionnaires, performed at six or 12 months after randomisation, and annually thereafter and (2) ad hoc reports of outcome events by local researchers at sites, general practitioners, and participants/carers. Reports of outcome events that were reported to the Trial Coordinating Centre are added to the trial database by trial staff. Trial staff reviewed these reports of outcome events, identified duplicate reports, and gathered information to support the characterisation of each event guided by the *RESTART reported event processing flowchart*): clinical records, death certificates, autopsy report (if performed), and brain imaging relating to any cerebral outcome events (local imaging report as well as the original imaging studies, which were independently reviewed by one of the consultant neuroradiologist members of the RESTART imaging panels). Trial staff redacted information that might identify participants or their antithrombotic drug use from these sources of information. These redacted documents were combined with a structured review form (*RESTART reported event processing checklist*) and given to a consultant neurologist medical assessor (Will Whiteley or Malcolm Macleod) for adjudication. A medical assessor classified the report of an outcome event as: (a) impossible to adjudicate (in which case trial staff obtained more information to enable subsequent adjudication), (b) an SAE/SUSAR, (c) a duplicate report of an outcome event, (d) not an outcome event, or (e) an outcome event (in which case they recorded an outcome event type and date of symptom onset). The outcome events that were confirmed after internal adjudication (e) were the only events that are used for analysis for reports for the Data Monitoring Committee and the final trial report. On 23 March 2018, the RESTART data manager (Jonathan Drever) exported all of the reported outcome events and internally adjudicated outcome events that had been categorised as the primary outcome in RESTART (recurrent intracerebral haemorrhage [ICH]). After removing duplicates and removing reports of outcome events that had not been internally adjudicated, he split the remaining events into three categories:

1. Reports of recurrent ICH that were not categorised as recurrent ICH after internal adjudication (n=10). We used all of these for external adjudication.
2. Reports of events that were not reported as recurrent ICH but were categorised as recurrent ICH after internal adjudication (n=5). We used all of these for external adjudication.
3. Reports of recurrent ICH that were categorised as recurrent ICH after internal adjudication (n=25). We took a random sample of ten of these events for external adjudication.

Dr Thomas Gattringer (consultant neurologist at Universitätsklinik für Neurologie, Medical University of Graz, Austria) was the independent external assessor during his visit to the Centre for Clinical Brain Sciences at the University of

Edinburgh in 2018. He reviewed the same information that had been provided to a medical assessor for internal adjudication, masked to the outcome of internal adjudication, and independently reviewed and categorised all 25 events (table overleaf). There was 96% agreement between the internal medical assessor and the external adjudicator on whether or not the 25 events were recurrent ICH.

Patient	Event	Reported event type	Classification by the internal adjudicator	Date of event	Classification by the external adjudicator	Agreement
87	213	Other Intracranial Haemorrhage	Recurrent ICH	24/06/2016	Recurrent ICH	yes
146	121	Other Intracranial Haemorrhage	Recurrent ICH	29/07/2016	Recurrent ICH	yes
12	45	Other Type of Stroke	Recurrent ICH	18/06/2015	Recurrent ICH	yes
153	168	Other Type of Stroke	Recurrent ICH	17/08/2015	Recurrent ICH	yes
156	112	Death	Recurrent ICH	01/02/2016	Death from frailty after index, not recurrent, ICH	no
56	32	Recurrent ICH	Ischaemic Stroke	18/08/2014	Ischaemic Stroke	yes
76	186	Recurrent ICH	Ischaemic Stroke	02/01/2017	Ischaemic Stroke	yes
89	42	Recurrent ICH	Not an Outcome Event	13/07/2015	Epileptic seizures	yes
89	41	Recurrent ICH	Not an Outcome Event	27/09/2015	Epileptic seizures	yes
96	188	Recurrent ICH	Not an Outcome Event	28/11/2017	Fall at home	yes
126	22	Recurrent ICH	Not an Outcome Event	02/02/2015	ICH before randomisation	yes
290	138	Recurrent ICH	Not an Outcome Event	18/04/2016	Death due to pre-randomisation ICH	yes
56	31	Recurrent ICH	Other Intracranial Haemorrhage	19/08/2014	Haemorrhagic transformation of ischaemic stroke	yes
370	171	Recurrent ICH	Other Intracranial Haemorrhage	27/01/2017	Convexity SAH	yes
452	192	Recurrent ICH	Other Intracranial Haemorrhage	31/10/2017	Convexity SAH	yes
26	11	Recurrent ICH	Recurrent ICH	16/01/2015	recurrent ICH	yes
79	149	Recurrent ICH	Recurrent ICH	22/09/2015	Recurrent ICH	yes
117	124	Recurrent ICH	Recurrent ICH	04/09/2015	Recurrent ICH	yes
124	76	Recurrent ICH	Recurrent ICH	28/03/2016	Recurrent ICH	yes
126	17	Recurrent ICH	Recurrent ICH	15/02/2015	Recurrent ICH	yes
126	51	Recurrent ICH	Recurrent ICH	17/04/2015	Recurrent ICH	yes
153	169	Recurrent ICH	Recurrent ICH	04/09/2015	Recurrent ICH	yes
189	67	Recurrent ICH	Recurrent ICH	31/12/2015	Recurrent ICH	yes
196	68	Recurrent ICH	Recurrent ICH	18/11/2015	Recurrent ICH	yes
336	177	Recurrent ICH	Recurrent ICH	14/11/2016	Recurrent ICH	yes

ICH = intracerebral haemorrhage; SAH = subarachnoid haemorrhage

Participant characteristics at randomisation by treatment allocation

	Start antiplatelet therapy (n=268)		Avoid antiplatelet therapy (n=269)	
Occlusive vascular diseases before intracerebral haemorrhage *				
Ischaemic heart disease	133	(50%)	110	(41%)
Ischaemic stroke	75	(28%)	88	(33%)
Transient ischaemic attack	57	(21%)	76	(28%)
Atrial fibrillation/flutter	42	(16%)	50	(19%)
Peripheral arterial disease	22	(8%)	14	(5%)
Valvular heart disease	11	(4%)	18	(7%)
Symptomatic venous thromboembolism	9	(3%)	10	(4%)
Stroke of uncertain pathological type	2	(<1%)	3	(1%)
Retinal artery occlusion	3	(<1%)	5	(2%)
Mesenteric ischaemia	1	(<1%)	1	(<1%)
Past history of haemorrhage before intracerebral haemorrhage				
Intracerebral haemorrhage	8	(3%)	11	(4%)
Gastrointestinal haemorrhage	7	(3%)	6	(2%)
Other type of intracranial haemorrhage	5	(2%)	6	(2%)
Other type of extracranial haemorrhage	3	(1%)	2	(1%)
Other relevant co-morbidities before intracerebral haemorrhage				
High blood pressure	194	(72%)	207	(77%)
Diabetes mellitus	57	(21%)	70	(26%)
Congestive cardiac failure	12	(5%)	8	(3%)
Renal failure on dialysis	3	(1%)	3	(1%)
Functional status[1]				
Able to lift both arms off the bed	242	(90%)	244	(91%)
Able to walk without the help of another person	199	(74%)	196	(73%)
Able to talk and not confused	234	(87%)	238	(89%)

Data are n (%). * Some participants had more than one co-morbidity.

Antithrombotic therapy before randomisation and during follow-up

	Start antiplatelet therapy (n=268)		Avoid antiplatelet therapy (n=269)	
**Antithrombotic therapy used before intracerebral haemorrhage**				
Antiplatelet therapy				
Aspirin monotherapy	132	(49%)	137	(51%)
Clopidogrel monotherapy	70	(26%)	63	(23%)
Aspirin and Clopidogrel	9	(3%)	5	(2%)
Aspirin and Dipyridamole	6	(2%)	5	(2%)
Other	4	(1%)	2	(1%)
Anticoagulant therapy				
Vitamin K antagonist	30	(11%)	41	(15%)
Non-vitamin K antagonist	8	(3%)	11	(4%)
Antiplatelet and anticoagulant therapy	9	(3%)	5	(2%)
**Preferred antiplatelet therapy that would be prescribed if allocated to start antiplatelet therapy ***				
Aspirin monotherapy	149	(56%)	150	(56%)
Clopidogrel monotherapy	117	(44%)	112	(42%)
Aspirin and Clopidogrel	1	(<1%)	5	(2%)
Dipyridamole monotherapy	1	(<1%)	1	(<1%)
Aspirin, Clopidogrel and Dipyridamole	0	(0%)	1	(<1%)
**Adherence to allocated treatment strategy before the first outcome event after randomisation §**				
Hospital/clinic discharge	259/265	(98%)	266/266	(99%)
First annual follow-up	193/218	(89%)	205/211	(97%)
Second annual follow-up	104/122	(85%)	105/113	(93%)
Third annual follow-up	59/71	(83%)	61/69	(88%)
Fourth annual follow-up	21/26	(81%)	20/24	(83%)

* Variable used in the minimisation algorithm. § Denominators indicate the number of participants surviving at each follow-up timepoint without a preceding outcome event

Anticoagulant therapy at discharge and during follow-up

	Start antiplatelet therapy (n=268)		Avoid antiplatelet therapy (n=269)	
Hospital/clinic discharge	1/268	(<1%)	1/269	(<1%)
First annual follow-up	8/265	(3%)	6/267	(2%)
Second annual follow-up	6/165	(4%)	5/161	(3%)
Third annual follow-up	9/103	(9%)	3/107	(3%)
Fourth annual follow-up	4/42	(10%)	1/39	(3%)

Denominators indicate the number of participants surviving at each follow-up timepoint

Blood pressure-lowering drug use at discharge and during follow-up, with average achieved blood pressures during follow-up, by treatment allocation

	**Start antiplatelet therapy**		**Avoid antiplatelet therapy**	
Hospital/clinic discharge	n=268		n=269	
None	53	(20%)	41	(15%)
One	82	(31%)	92	(34%)
Two	92	(34%)	84	(31%)
Three	30	(11%)	36	(13%)
Four or more	11	(4%)	16	(6%)
First annual follow-up	n=265		n=267	
Median systolic blood pressure, mmHg	132	(120-140)	130	(121-140)
Median diastolic blood pressure, mmHg	74	(70-80)	74	(69-80)
None	46	(17%)	34	(13%)
One	54	(20%)	78	(29%)
Two	85	(32%)	75	(28%)
Three	56	(21%)	50	(19%)
Four or more	24	(9%)	30	(11%)
Second annual follow-up	n=165		n=161	
Median systolic blood pressure, mmHg	129	(120-138)	130	(120-140)
Median diastolic blood pressure, mmHg	72	(66-80)	72	(67-80)
None	29	(18%)	27	(17%)
One	39	(24%)	34	(21%)
Two	41	(25%)	48	(30%)
Three	34	(21%)	33	(21%)
Four or more	10	(10%)	11	(10%)
Third annual follow-up	n=103		n=107	
Median systolic blood pressure, mmHg	133	(120-141)	131	(120-140)
Median diastolic blood pressure, mmHg	76	(70-82)	72	(68-80)
None	22	(21%)	21	(20%)
One	25	(24%)	19	(18%)
Two	23	(22%)	31	(29%)
Three	23	(22%)	25	(23%)
Four or more	10	(10%)	11	(10%)
Fourth annual follow-up	n=42		n=39	
Median systolic blood pressure, mmHg	132	(120-140)	129	(120-139)
Median diastolic blood pressure, mmHg	72	(62-80)	74	(63-80)
None	9	(21%)	3	(8%)
One	11	(26%)	11	(28%)
Two	15	(36%)	9	(23%)
Three	7	(17%)	12	(31%)
Four or more	0	(0%)	4	(10%)

Data are n (%) or median (inter-quartile range).

Cumulative absolute risks, and risk differences, of the primary outcome and key secondary outcomes, by treatment allocation and by year

	Start antiplatelet therapy (n=268)	Avoid antiplatelet therapy (n=268)	Risk difference
Recurrent symptomatic spontaneous intracerebral haemorrhage			
Year 1	3·1 (1·6 to 6·2)	7·3 (4·6 to 11·4)	-4·2 (-8·1 to -0·3)
Year 2	5·5 (3·1 to 9·7)	10·3 (6·9 to 15·2)	-4·8 (-9·9 to 0·4)
Year 3	5·5 (3·1 to 9·7)	10·3 (6·9 to 15·2)	-4·8 (-9·9 to 0·4)
Year 4	5·5 (3·1 to 9·7)	10·3 (6·9 to 15·2)	-4·8 (-9·9 to 0·4)
All major haemorrhagic events (all types of symptomatic spontaneous or traumatic intracranial haemorrhage, or symptomatic major extracranial haemorrhage)			
Year 1	5·2 (3·0 to 8·8)	8·0 (5·3 to 12·2)	-2·9 (-7·2 to 1·5)
Year 2	7·6 (4·7 to 12·0)	11·0 (7·5 to 16·0)	-3·5 (-8·9 to 2·0)
Year 3	7·6 (4·7 to 12·0)	11·0 (7·5 to 16·0)	-3·5 (-8·9 to 2·0)
Year 4	7·6 (4·7 to 12·0)	11·0 (7·5 to 16·0)	-3·5 (-8·9 to 2·0)
All major occlusive vascular events (symptomatic ischaemic stroke, myocardial infarction, mesenteric ischaemia, peripheral arterial occlusion, deep vein thrombosis, pulmonary embolism, or carotid/coronary/peripheral arterial revascularisation procedures)			
Year 1	7·6 (4·9 to 11·8)	9·6 (6·6 to 14·1)	-2·0 (-7·1 to 3·0)
Year 2	14·2 (10·0 to 20·0)	14·0 (9·9 to 19·5)	0·2 (-6·6 to 7·1)
Year 3	19·0 (13·6 to 26·1)	19·2 (13·7 to 26·4)	-0·2 (-9·0 to 8·6)
Year 4	30·9 (21·3 to 43·4)	23·9 (16·4 to 33·9)	7·0 (-7·0 to 21·0)
All major haemorrhagic or occlusive vascular events			
Year 1	12·7 (9·1 to 17·6)	17·1 (12·9 to 22·4)	-4·4 (-10·7 to 1·9)
Year 2	21·4 (16·3 to 27·7)	23·7 (18·5 to 30·0)	-2·3 (-10·3 to 5·7)
Year 3	24·0 (18·5 to 30·9)	28·6 (22·4 to 36·0)	-4·6 (-13·7 to 4·6)
Year 4	33·1 (24·2 to 44·2)	33·3 (25·2 to 43·1)	-0·1 (-13·6 to 13·3)
All major occlusive vascular events (protocol-defined)			
Year 1	9·5 (6·4 to 14·0)	14·4 (10·6 to 19·6)	-4·9 (-10·8 to 0·9)
Year 2	18·6 (13·8 to 24·9)	20·6 (15·7 to 26·8)	-2·0 (-9·8 to 5·9)
Year 3	22·4 (16·8 to 29·7)	25·7 (19·7 to 33·3)	-3·3 (-12·7 to 6·0)
Year 4	29·9 (21·2 to 41·1)	30·5 (22·5 to 40·6)	-0·6 (-14·1 to 12·8)
All major vascular events (protocol-defined)			
Year 1	8·8 (5·9 to 13·1)	16·7 (12·6 to 22·0)	-7·9 (-13·7 to -2·1)
Year 2	16·2 (11·8 to 22·0)	25·6 (20·2 to 32·1)	-9·3 (-17·1 to -1·5)
Year 3	21·5 (16·0 to 28·6)	31·1 (24·7 to 38·7)	-9·6 (-19·0 to -0·2)
Year 4	30·2 (21·6 to 41·1)	35·5 (27·5 to 45·1)	-5·4 (-18·5 to 7·8)

Kaplan-Meier plot showing the risk of the first occurrence of any major haemorrhagic event

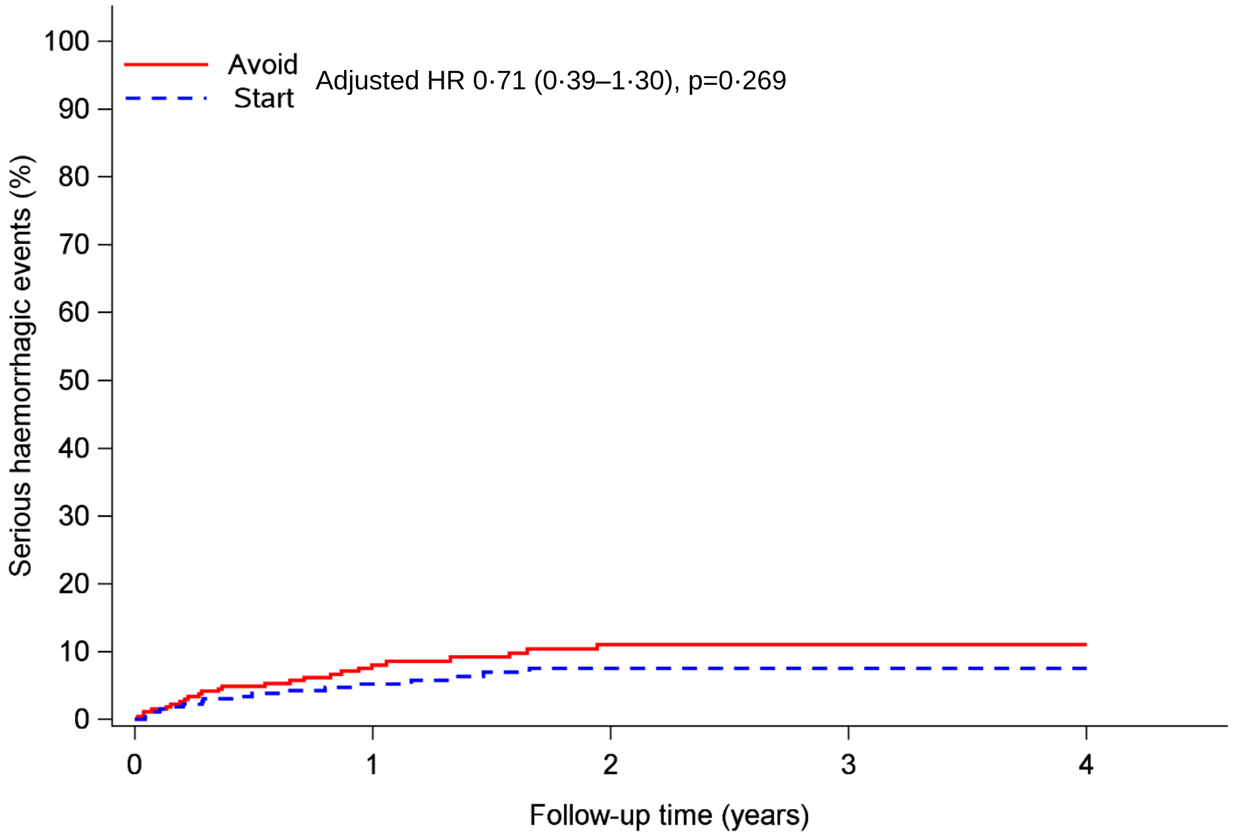

Patients-at-Risk (No. Cumulative Events)

	0	1	2	3	4
Avoid	268 (0)	184 (20)	121 (25)	73 (25)	22 (25)
Start	268 (0)	188 (13)	120 (17)	70 (17)	24 (17)

Numbers below the x-axis indicate numbers of survivors under follow-up at the start of each year (and the cumulative number of participants with a first event in follow-up) according to treatment allocation

Kaplan-Meier plot showing the risk of the first occurrence of any major occlusive vascular event

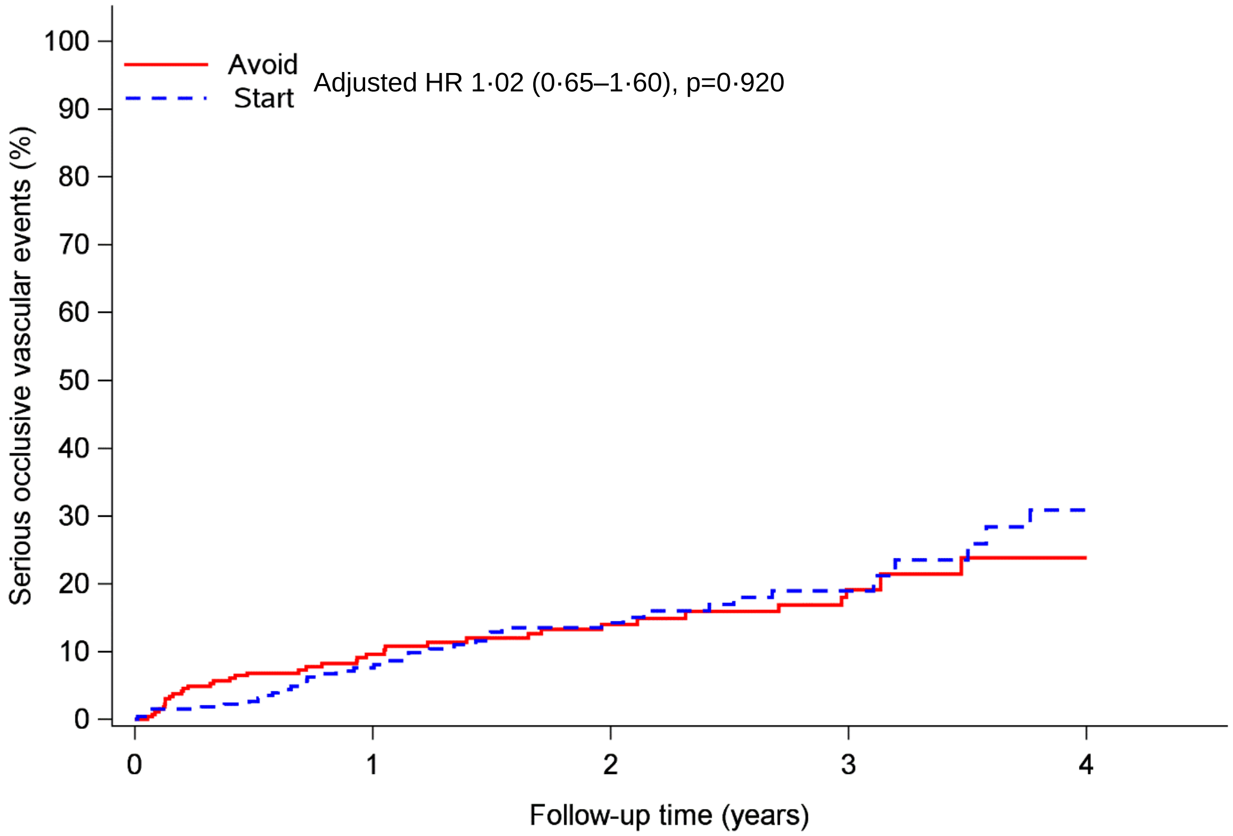

Patients-at-Risk (No. Cumulative Events)

Avoid	268 (0)	172 (24)	111 (31)	66 (36)	18 (38)
Start	268 (0)	181 (18)	111 (29)	62 (34)	18 (39)

Numbers below the x-axis indicate numbers of survivors under follow-up at the start of each year (and the cumulative number of participants with a first event in follow-up) according to treatment allocation

Kaplan-Meier plot showing the risk of the first occurrence of any major haemorrhagic or occlusive vascular event

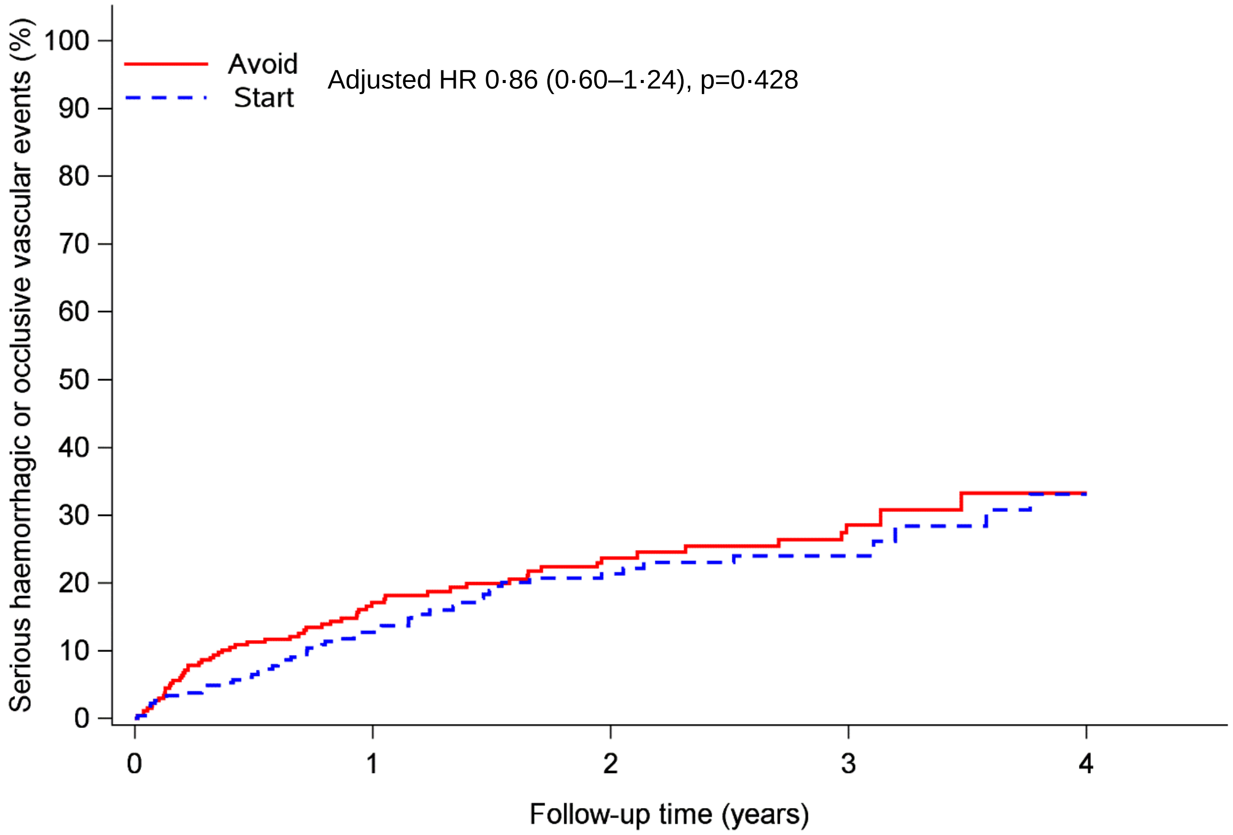

Patients-at-Risk (No. Cumulative Events)

Avoid	268 (0)	166 (43)	103 (54)	62 (59)	17 (61)
Start	268 (0)	177 (31)	107 (46)	60 (49)	18 (53)

Numbers below the x-axis indicate numbers of survivors under follow-up at the start of each year (and the cumulative number of participants with a first event in follow-up) according to treatment allocation

Kaplan-Meier plot showing the risk of the first occurrence of the composite outcome of major vascular events, as proposed in the trial protocol

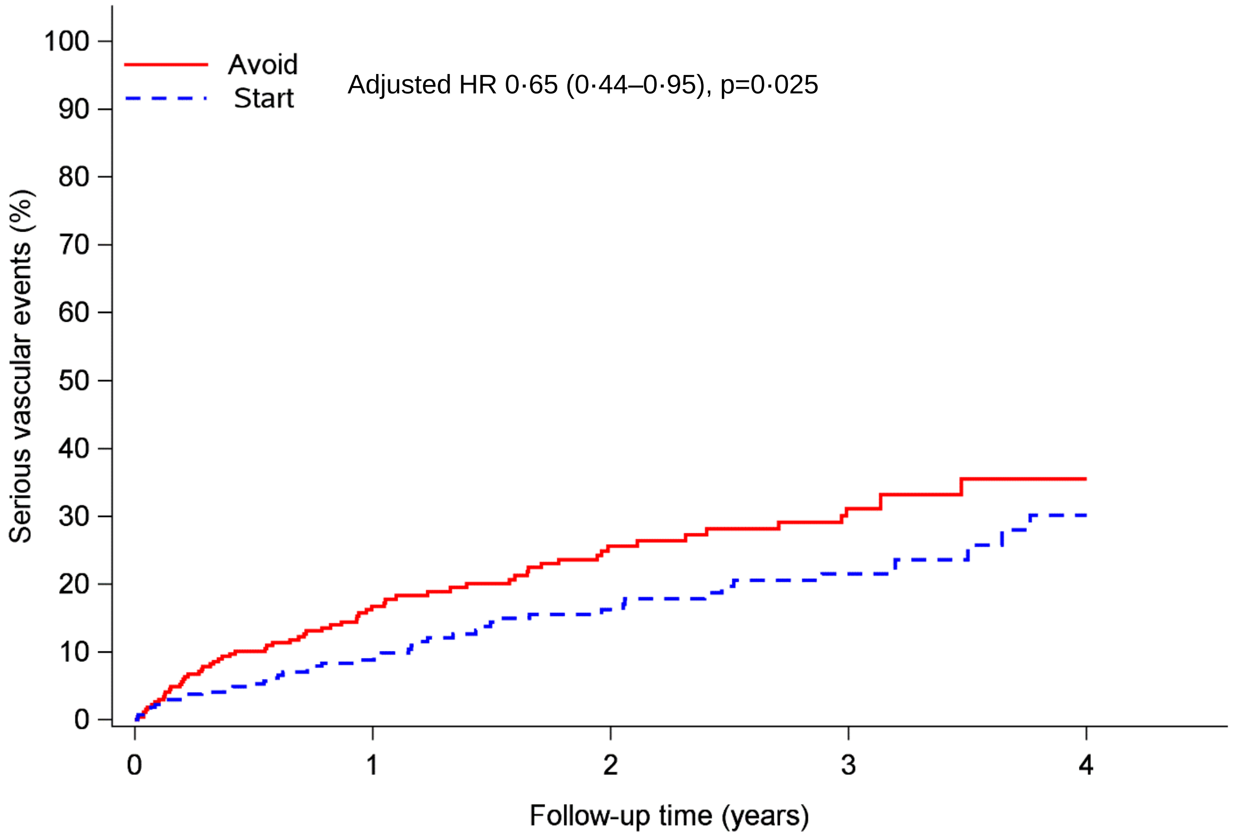

Patients-at-Risk (No. Cumulative Events)

Avoid	268 (0)	169 (42)	105 (57)	63 (63)	18 (65)
Start	268 (0)	185 (22)	115 (35)	66 (41)	21 (45)

Numbers below the x-axis indicate numbers of survivors under follow-up at the start of each year (and the cumulative number of participants with a first event in follow-up) according to treatment allocation

Pre-specified exploratory sub-group analyses of all major haemorrhagic events

Subgroup	Start antiplatelet drugs [Events/N(%)]	Avoid antiplatelet drugs [Events/N(%)]	Adjusted Hazard Ratio (95% CI)	Test of interaction p-value
Intracerebral haemorrhage location				
Lobar	13/166 (7.8%)	12/166 (7.2%)	1.10 (0.50 to 2.42)	.096
Non-Lobar	5/102 (4.9%)	13/102 (12.7%)	0.36 (0.13 to 1.02)	
Time since intracerebral haemorrhage symptom onset				
<= median time from symptom onset	10/129 (7.8%)	14/140 (10.0%)	0.74 (0.33 to 1.66)	.892
> median time from symptom onset	8/139 (5.8%)	11/128 (8.6%)	0.68 (0.27 to 1.69)	
Antiplatelet drug(s) that the patient's clinician would start				
Aspirin	12/149 (8.1%)	13/149 (8.7%)	0.89 (0.40 to 1.94)	.393
Other	6/119 (5.0%)	12/119 (10.1%)	0.51 (0.19 to 1.37)	
Participant's age at randomisation				
<70 years	2/73 (2.7%)	6/73 (8.2%)	0.33 (0.07 to 1.66)	.307
>=70 years	16/195 (8.2%)	19/195 (9.7%)	0.83 (0.43 to 1.61)	
Predicted probability of good outcome at 6 months				
<0.15	3/48 (6.3%)	8/51 (15.7%)	0.38 (0.10 to 1.44)	.284
>=0.15	15/220 (6.8%)	17/217 (7.8%)	0.86 (0.43 to 1.73)	
History of atrial fibrillation				
No	11/207 (5.3%)	16/195 (8.2%)	0.66 (0.31 to 1.43)	.74
Yes	7/61 (11.5%)	9/73 (12.3%)	0.82 (0.30 to 2.21)	
Type of antithrombotic drug regimen before intracerebral haemorrhage				
Anticoagulant with/without antiplatelet	5/47 (10.6%)	8/57 (14.0%)	0.69 (0.22 to 2.14)	.931
Antiplatelet alone	13/221 (5.9%)	17/211 (8.1%)	0.73 (0.36 to 1.51)	
Overall	18/268 (6.7%)	25/268 (9.3%)	0.71 (0.39 to 1.30)	

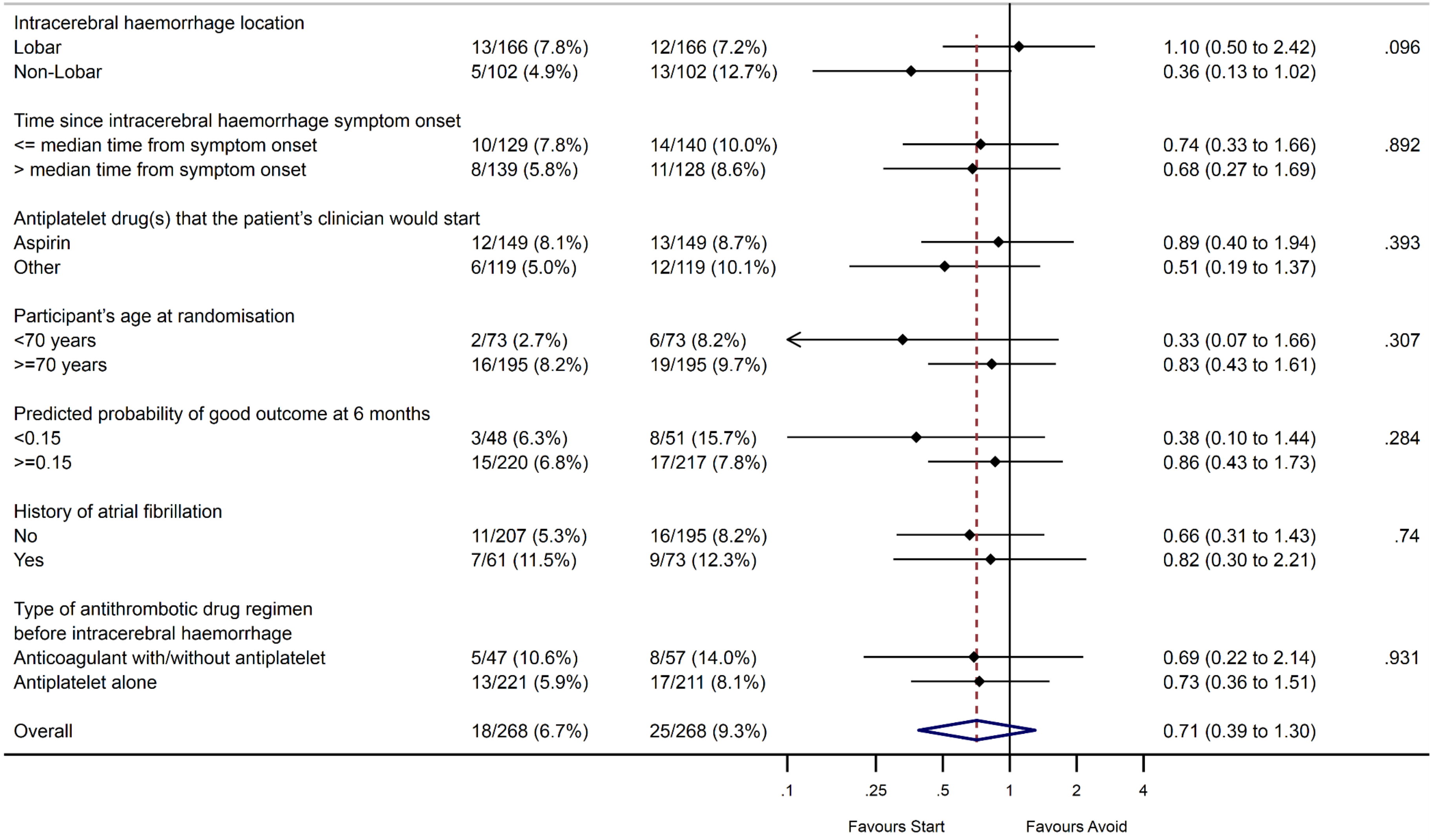

Pre-specified exploratory sub-group analyses of all major occlusive vascular events

Subgroup	Start antiplatelet drugs [Events/N(%)]	Avoid antiplatelet drugs [Events/N(%)]	Adjusted Hazard Ratio (95% CI)	Test of interaction p-value
Intracerebral haemorrhage location				
Lobar	19/166 (11.4%)	23/166 (13.9%)	0.78 (0.42 to 1.43)	.197
Non-Lobar	20/102 (19.6%)	15/102 (14.7%)	1.42 (0.72 to 2.80)	
Time since intracerebral haemorrhage symptom onset				
<= median time from symptom onset	24/129 (18.6%)	20/140 (14.3%)	1.35 (0.74 to 2.46)	.189
> median time from symptom onset	15/139 (10.8%)	18/128 (14.1%)	0.73 (0.37 to 1.46)	
Antiplatelet drug(s) that the patient's clinician would start				
Aspirin	24/149 (16.1%)	25/149 (16.8%)	0.98 (0.56 to 1.71)	.785
Other	15/119 (12.6%)	13/119 (10.9%)	1.11 (0.53 to 2.35)	
Participant's age at randomisation				
<70 years	9/73 (12.3%)	7/73 (9.6%)	1.44 (0.53 to 3.90)	.445
>=70 years	30/195 (15.4%)	31/195 (15.9%)	0.94 (0.57 to 1.55)	
Predicted probability of good outcome at 6 months				
<0.15	12/48 (25.0%)	8/51 (15.7%)	1.52 (0.62 to 3.74)	.31
>=0.15	27/220 (12.3%)	30/217 (13.8%)	0.89 (0.53 to 1.50)	
History of atrial fibrillation				
No	28/207 (13.5%)	20/195 (10.3%)	1.40 (0.79 to 2.49)	.086
Yes	11/61 (18.0%)	18/73 (24.7%)	0.61 (0.28 to 1.30)	
Type of antithrombotic drug regimen before intracerebral haemorrhage				
Anticoagulant with/without antiplatelet	9/47 (19.1%)	14/57 (24.6%)	0.73 (0.31 to 1.69)	.315
Antiplatelet alone	30/221 (13.6%)	24/211 (11.4%)	1.21 (0.71 to 2.08)	
Overall	39/269 (14.5%)	38/268 (13.4%)	1.02 (0.65 to 1.60)	

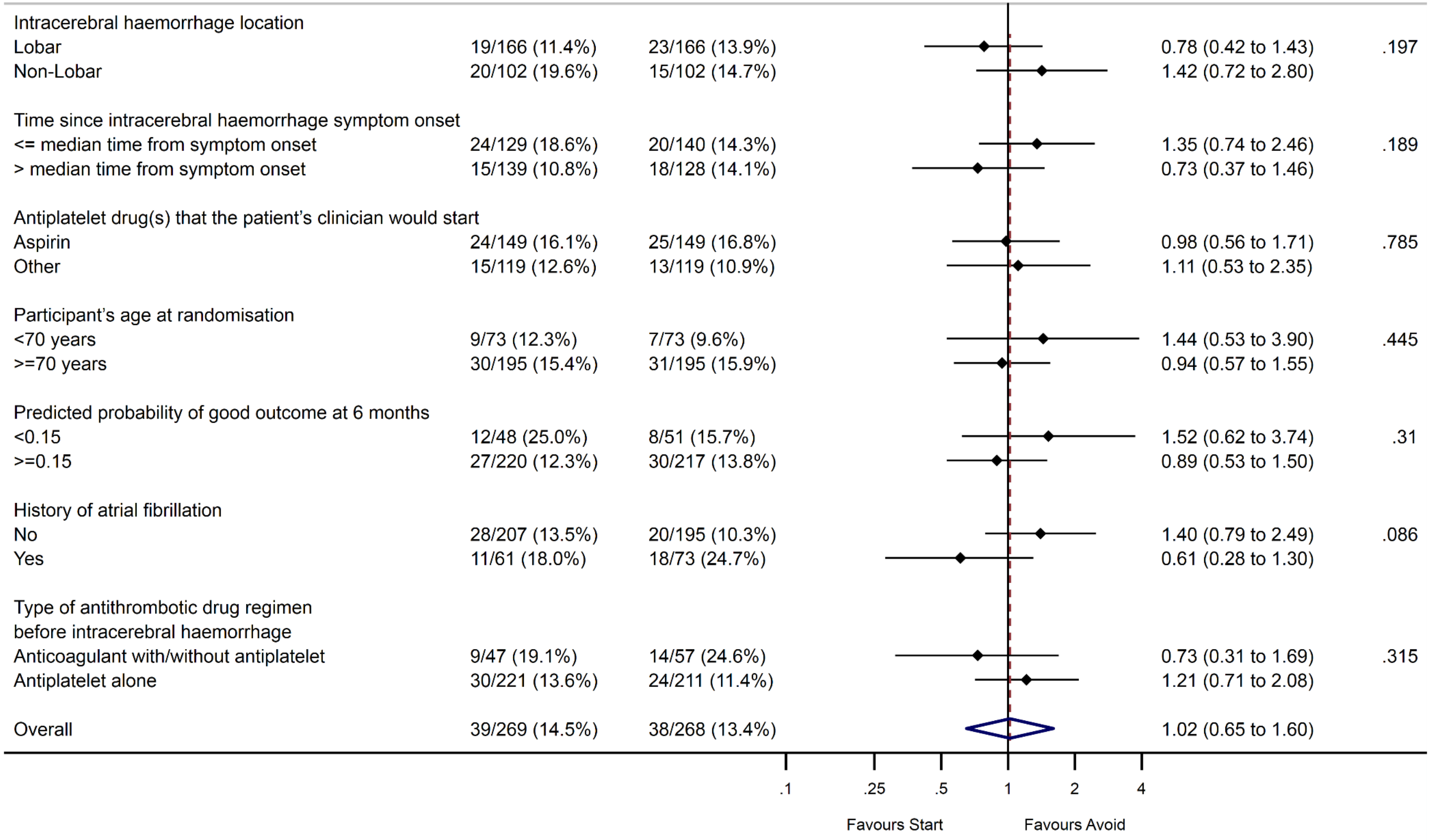

Pre-specified exploratory sub-group analyses of the composite outcome of all major haemorrhagic or occlusive vascular events

Subgroup	Start antiplatelet drugs [Events/N(%)]	Avoid antiplatelet drugs [Events/N(%)]	Adjusted Hazard Ratio (95% CI)	Test of interaction p-value
Intracerebral haemorrhage location				
Lobar	30/166 (18.1%)	34/166 (20.5%)	0.86 (0.52 to 1.41)	.987
Non-Lobar	24/102 (23.5%)	27/102 (26.5%)	0.87 (0.50 to 1.51)	
Time since intracerebral haemorrhage symptom onset				
<= median time from symptom onset	32/129 (24.8%)	33/140 (23.6%)	1.05 (0.64 to 1.71)	.258
> median time from symptom onset	22/139 (15.8%)	28/128 (21.9%)	0.68 (0.39 to 1.19)	
Antiplatelet drug(s) that the patient's clinician would start				
Aspirin	34/149 (22.8%)	37/149 (24.8%)	0.89 (0.56 to 1.43)	.813
Other	20/119 (16.8%)	24/119 (20.2%)	0.81 (0.45 to 1.48)	
Participant's age at randomisation				
<70 years	11/73 (15.1%)	13/73 (17.8%)	0.92 (0.41 to 2.07)	.849
>=70 years	43/195 (22.1%)	48/195 (24.6%)	0.85 (0.56 to 1.28)	
Predicted probability of good outcome at 6 months				
<0.15	14/48 (29.2%)	16/51 (31.4%)	0.86 (0.42 to 1.76)	.984
>=0.15	40/220 (18.2%)	45/217 (20.7%)	0.86 (0.56 to 1.32)	
History of atrial fibrillation				
No	38/207 (18.4%)	36/195 (18.5%)	1.03 (0.65 to 1.63)	.205
Yes	16/61 (26.2%)	25/73 (34.2%)	0.62 (0.33 to 1.17)	
Type of antithrombotic drug regimen before intracerebral haemorrhage				
Anticoagulant with/without antiplatelet	13/47 (27.7%)	20/57 (35.1%)	0.71 (0.35 to 1.44)	.488
Antiplatelet alone	41/221 (18.6%)	41/211 (19.4%)	0.95 (0.62 to 1.47)	
Overall	54/268 (20.1%)	61/268 (22.8%)	0.86 (0.60 to 1.24)	

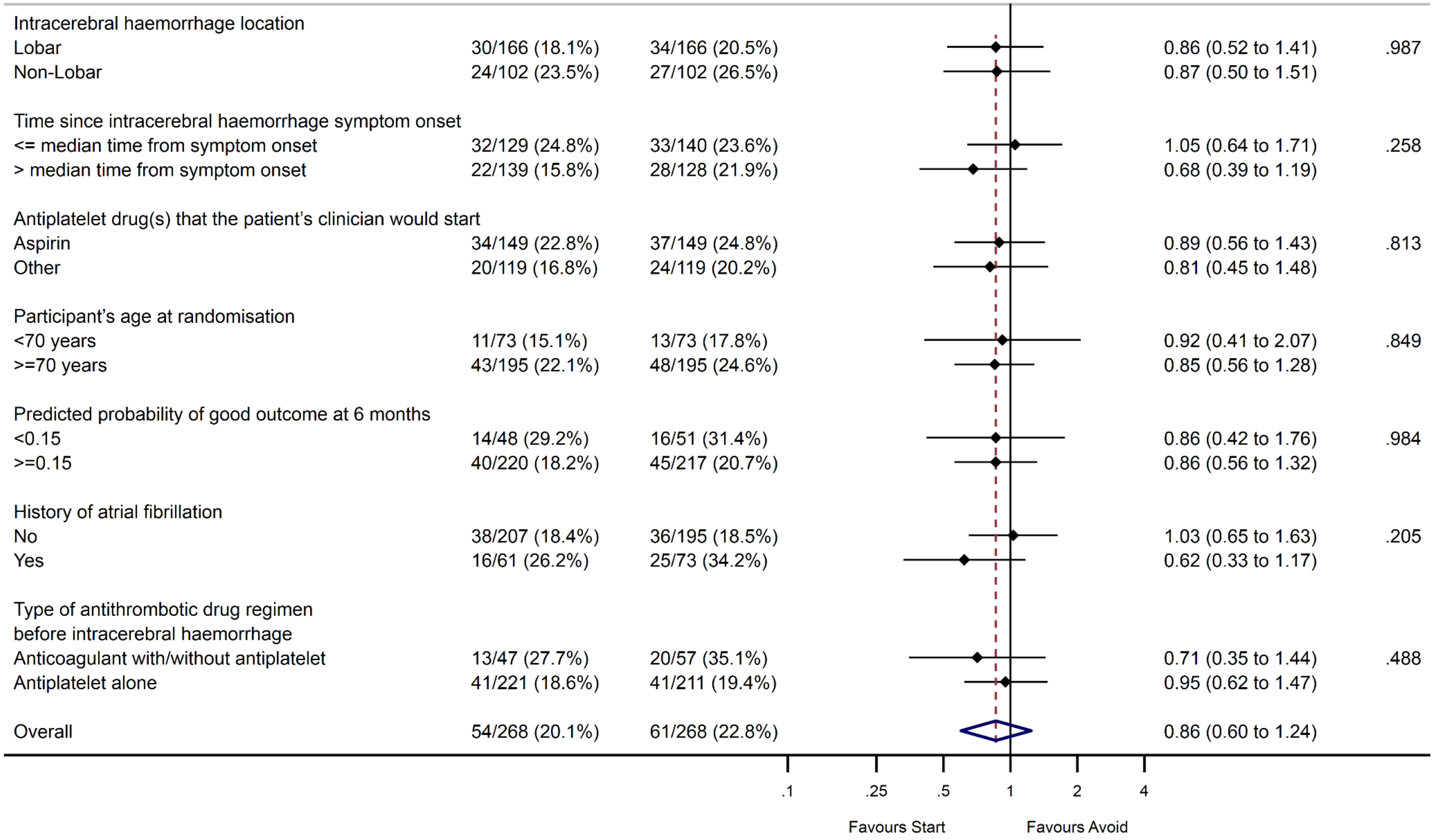

modified Rankin Scale (mRS) scores rated at randomisation by collaborators and during follow-up by participants/carers,[2-4] by treatment allocation

	Start antiplatelet therapy		Avoid antiplatelet therapy	
At randomisation	n=268		n=269	
0 (no symptoms)	31	(12%)	26	(10%)
1 (no significant disability)	66	(25%)	59	(22%)
2 (slight disability)	47	(17%)	62	(23%)
3 (moderate disability)	55	(21%)	53	(20%)
4 (moderately severe disability)	57	(21%)	50	(18%)
5 (severe disability)	12	(4%)	19	(7%)
First annual follow-up (p=0·920)	n=230		n=231	
0 (no symptoms)	34	(15%)	36	(16%)
1 (no significant disability)	41	(18%)	36	(16%)
2 (slight disability)	20	(9%)	28	(12%)
3 (moderate disability)	75	(33%)	72	(31%)
4 (moderately severe disability)	21	(9%)	13	(6%)
5 (severe disability)	16	(7%)	24	(10%)
6 (dead)	23	(10%)	22	(10%)
Second follow-up (p=0·408)	n=176		n=178	
0 (no symptoms)	19	(11%)	27	(15%)
1 (no significant disability)	21	(12%)	22	(12%)
2 (slight disability)	16	(9%)	20	(11%)
3 (moderate disability)	63	(36%)	52	(29%)
4 (moderately severe disability)	11	(6%)	8	(5%)
5 (severe disability)	15	(9%)	17	(10%)
6 (dead)	31	(18%)	32	(18%)
Third annual follow-up (p=0·328)	n=118		n=121	
0 (no symptoms)	13	(11%)	12	(10%)
1 (no significant disability)	7	(6%)	16	(13%)
2 (slight disability)	11	(9%)	17	(14%)
3 (moderate disability)	41	(35%)	31	(26%)
4 (moderately severe disability)	7	(6%)	6	(5%)
5 (severe disability)	8	(7%)	10	(8%)
6 (dead)	31	(26%)	29	(24%)
Fourth annual follow-up (p=0·783)	n=49		n=48	
0 (no symptoms)	4	(8%)	5	(10%)
1 (no significant disability)	4	(8%)	5	(10%)
2 (slight disability)	2	(4%)	4	(8%)
3 (moderate disability)	19	(38%)	13	(27%)
4 (moderately severe disability)	2	(4%)	1	(2%)
5 (severe disability)	4	(8%)	5	(10%)
6 (dead)	15	(30%)	15	(31%)

The analysis for each year is restricted to participants who were randomised at least the same number of years before the end of recruitment, to avoid including early deaths in the relevant follow-up year when the corresponding surviving recruits would not have had the potential to be assessed.

Serious adverse events, classified by organ system

MedDRA system organ class	Start antiplatelet therapy (n=268)	Avoid antiplatelet therapy (n=269)
Blood and lymphatic system disorders	n=0	n=0
Cardiac disorders	n=2	n=0
Congenital, familial and genetic disorders	n=0	n=0
Ear and labyrinth disorders	n=0	n=0
Endocrine disorders	n=0	n=0
Eye disorders	n=0	n=0
Gastrointestinal disorders	n=0	n=0
General disorders and administration site conditions	n=0	n=0
Hepatobiliary disorders	n=0	n=1
Immune system disorders	n=0	n=0
Infections and infestations	n=0	n=0
Injury, poisoning and procedural complications	n=1	n=1
Investigations	n=0	n=0
Metabolism and nutrition disorders	n=0	n=0
Musculoskeletal and connective tissue Disorders	n=1	n=1
Neoplasms benign, malignant and unspecified (including cysts and polyps)	n=0	n=1
Nervous system disorders	n=1	n=1
Pregnancy, puerperium and perinatal Conditions	n=0	n=0
Psychiatric disorders	n=0	n=0
Renal and urinary disorders	n=0	n=0
Reproductive system and breast disorders	n=0	n=0
Respiratory, thoracic and mediastinal disorders	n=0	n=0
Skin and subcutaneous tissue disorders	n=0	n=0
Social circumstances	n=0	n=0
Surgical and medical procedures	n=1	n=0
Vascular disorders	n=0	n=0

References

1. Counsell C, Dennis M, McDowall M, Warlow C. Predicting outcome after acute and subacute stroke: development and validation of new prognostic models. *Stroke* 2002; **33**(4): 1041-7.
2. Bruno A, Akinwuntan AE, Lin C, et al. Simplified modified rankin scale questionnaire: reproducibility over the telephone and validation with quality of life. *Stroke* 2011; **42**(8): 2276-9.
3. Bruno A, Shah N, Lin C, et al. Improving modified Rankin Scale assessment with a simplified questionnaire. *Stroke* 2010; **41**(5): 1048-50.
4. Dennis M, Mead G, Doubal F, Graham C. Determining the modified Rankin score after stroke by postal and telephone questionnaires. *Stroke* 2012; **43**(3): 851-3.
